# Dark- and Photoexcited-State Electronic Structures of [Ru(SO_2_)(NH_3_)_4_X]Y Complexes and Their Relation to SO_2_ Photoconversion Efficiency

**DOI:** 10.1021/acs.jpclett.6c02623

**Published:** 2026-09-24

**Authors:** Haoqian Zhou, Jacqueline M. Cole

**Affiliations:** Ray Dolby Centre, Cavendish Laboratory, Department of Physics, 2152 University of Cambridge, J. J. Thomson Avenue, Cambridge CB3 0US, U.K.

## Abstract

Single crystals of the [Ru­(SO_2_)­(NH_3_)_4_X]Y family of complexes are promising photoswitches or photoactuators; they respond structurally to visible light, undergoing SO_2_ linkage photoisomerization with high spatiotemporal precision. However, the SO_2_ photoconversion efficiency (PCE) varies markedly with the *trans*-ligand X and no unifying molecular design principle is yet available. This study addresses this knowledge gap by performing electronic-structure calculations on 11 respective crystal structures of these complexes using reference crystal structures characterized by single-crystal X-ray diffraction under light irradiation. We focus on 4 structural archetypes of [Ru­(SO_2_)­(NH_3_)_4_X]­[tosylate]_2_, where X = H_2_O (**1**), 3-F-pyridine (**2**), 3-Cl-pyridine (**3**), and 3-Br-pyridine (**4**), to elucidate how the *trans*-ligands control the Ru-centered electronic rearrangement upon photoexcitation. The dark-state projected densities of states of these crystal structures are qualitatively similar across the series, exhibiting [Ru]-S π-bonding and [Ru]-X π/σ interactions. By contrast, electronic-structure calculations using time-dependent density functional theory reveal distinct photoexcited-state behavior: in **1**, photoexcitation populates an antibonding *d*_*yz*_-*p*_*y*_ orbital, weakening the [Ru]-S bond and lowering the SO_2_ photoisomerization barrier; in **2**−**4**, photoexcitation simultaneously populates this antibonding orbital and depopulates its bonding counterpart, weakening the [Ru]-S bond substantially more to afford a correspondingly higher PCE than in **1**. Response charge-density analyses visualize the corresponding photoinduced charge redistribution in real space, consistent with the finer ordering of the PCE: **3** < **2** < **4**. We additionally propose the Ru *d*-band center as a descriptor for PCE: within a common class of *trans*-ligand, the PCE tends to decrease as the *d*-band center shifts toward the Fermi level, as shown here for ten pyridyl-ligated structures, although other factors also contribute to the photoisomerization process. Together, these results provide a mechanistic account of photoexcited-state changes in the electronic structure of [Ru­(SO_2_)­(NH_3_)_4_X]Y complexes and suggest a practical screening metric for the rational molecular design of this family of high-PCE photoresponsive single crystals for quantum photosensing applications.

## Graphical abstract

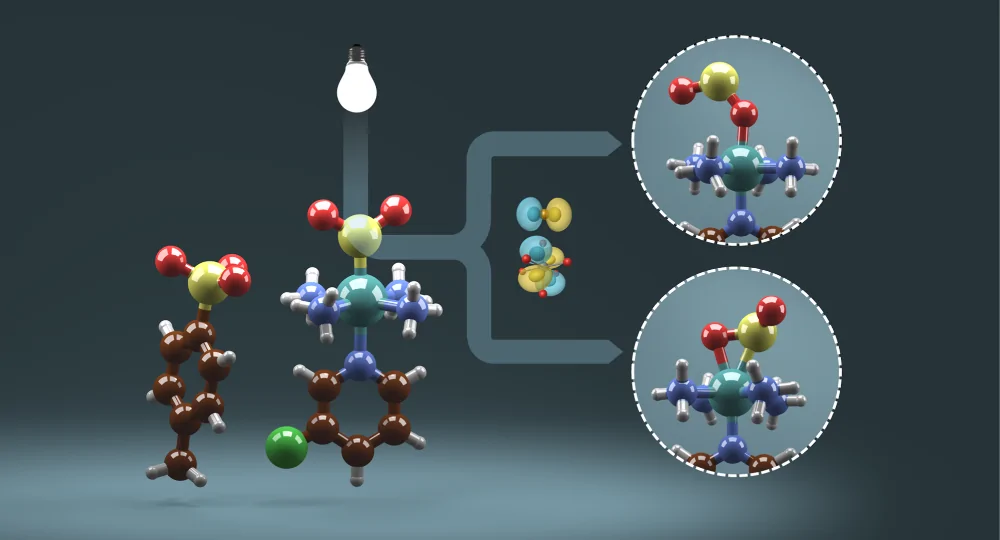

Precise manipulation of micro- and nanoscale chemical structures under external stimuli has long been a central theme of materials chemistry. Results have led to diverse applications that span biomedicine, nanotechnology, and beyond.^1–3^ When their single-crystal forms are actuated by external stimuli such as heat,^4^ light,^5^ magnetic field,^6^ electric field,^7^ or pH,^8^ certain types of materials can exhibit a wide range of remarkable behaviors, including photoresponsive mechanical motions,^9^ crystalline deformations,^10,11^ and changes in optical appearance.^12,13^ These phenomena have been harnessed via the construction of molecular machines,^14^ data storage devices,^15^ and next-generation quantum computers.^16^ Among the various external stimuli used to drive actuation, light offers distinct advantages, including a clean process, high spatial and temporal precision, remote controllability, and tunable interaction with matter.^17^ Over the past few decades, a broad range of organic,^4,18^ inorganic,^19^ and hybrid materials^20,21^ have emerged as promising light-driven actuators, demonstrating diverse motion functionalities and excellent stabilities.

Owing to their broadband light absorption and distinctive SO_2_ linkage photoisomerization, photoresponsive single crystals of [Ru­(SO_2_)­(NH_3_)_4_X]Y complexes (where X denotes the *trans*-ligand and Y the counterion) have attracted increasing interest as promising candidates for optical machines, data storage media, and quantum sensing.^11,22,23^ In contrast to many organic molecular photoswitches and photoactuators that require UV excitation, single crystals of [Ru­(SO_2_)­(NH_3_)_4_X]Y complexes can be photoexcited by visible light through their intrinsic metal-to-ligand charge-transfer (MLCT) and metal-centered (MC) transitions.^11,24^ This avoids the potentially damaging effects of UV irradiation on materials or their hosts and broadens their applicability.

The structural features common to this family of complexes are illustrated in Figure 1. The Ru center adopts an octahedral coordination geometry in which four ammine ligands occupy the equatorial plane, while the two axial positions are held by the photoactive SO_2_ ligand and the *trans*-ligand X, which therefore compete for the same Ru *d* orbitals along the S–Ru–X axis. The resulting complex cation carries a charge of m+ that is balanced by n– counterion Y, which occupy the interstitial regions of the lattice and define the cavity available to the SO_2_ ligand. Under suitable photoexcitation conditions, the photoactive SO_2_ ligand undergoes linkage photoisomerization, in which the dark state (S-bound configuration η^1^-SO_2_) is converted into one or both of two metastable Ru-coordinated configurations: O-bound η^1^-OSO (MS1) and (O–S) side-bound η^2^-(OS)O (MS2).^22^ Both metastable states revert thermally to the dark state, so that the photoswitching cycle is fully reversible. The photoconversion efficiency (PCE) of these SO_2_ metastable states is governed primarily by the nature of the *trans*-ligand coordinated to Ru, while steric constraints imposed by the counterion can exert secondary effects.^23,25^ The PCE is determined by photocrystallography: single-crystal X-ray diffraction data are collected at low temperature postlight irradiation, and the fractions of the metastable configurations are obtained from the refined site occupancies of the SO_2_ ligand.^22^ To date, members of this Ru-based family have been reported with PCEs ranging from 10.4(1)% to 100%,^10,11,22–35^ although complete photoconversion has so far been demonstrated only in these complexes: [Ru­(SO_2_)­(NH_3_)_4_(3-I-pyridine)]­[tosylate]_2_,^29^ [Ru­(SO_2_)­(NH_3_)_4_(3-Br-pyridine)]­[tosylate]_2_,^26^ and [Ru­(SO_2_)­(NH_3_)_4_(4-Br-pyridine)]­[tosylate]_2_.^11^ While single crystals of all photoresponsive [Ru­(SO_2_)­(NH_3_)_4_X]Y complexes can, in principle, function as optical switches through the controllable generation of two metastable states, only a few exhibit photoswitching with concomitant molecular transduction within the crystal lattice which occurs via intermolecular interactions between the cationic complex and anionic counterions.^22,24,26^

**Figure 1:**
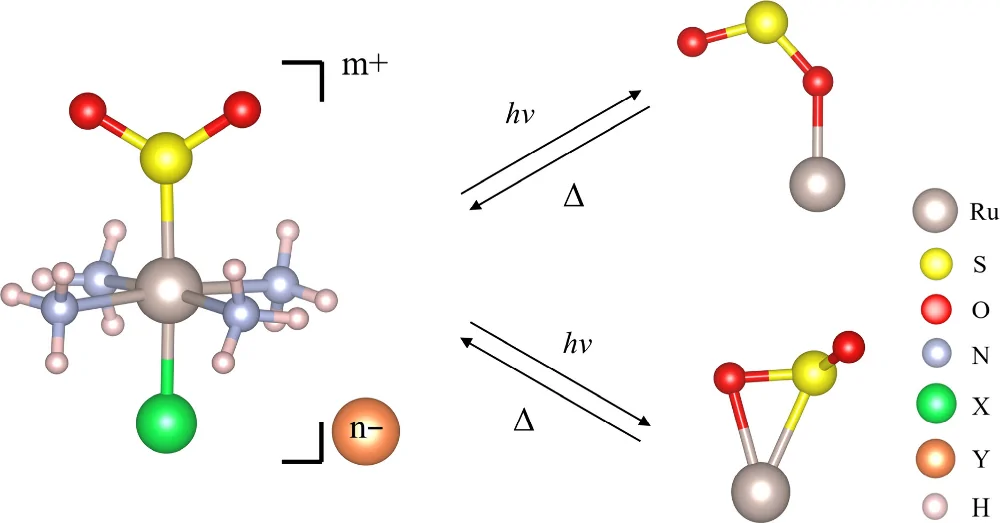
Structure of the [Ru­(SO_2_)­(NH_3_)_4_X]Y family of complexes. The Ru center is coordinated by four equatorial ammine ligands, with the photoactive SO_2_ ligand and the *trans*-ligand X occupying the two axial sites; the complex cation of charge m+ is balanced by n– counterions Y. Photoexcitation (*hν*) converts the S-bound η^1^-SO_2_ dark state into the O-bound η^1^-OSO (MS1, top) and side-bound η^2^-(OS)O (MS2, bottom) metastable configurations, both of which revert thermally (Δ) to the dark state.

The rational design of photoresponsive materials with high PCE demands the identification of reliable molecular descriptors. Although experimental advances in [Ru­(SO_2_)­(NH_3_)_4_X]Y complexes have demonstrated the feasibility of tuning functionality at the molecular scale, the search for high-PCE candidates remains dominated by trial-and-error approaches, owing to the absence of a universal design principle. Several empirically determined insights have been proposed for these Ru-based complexes from photocrystallographic results. One insight links PCE to a positive correlation between the σ-donor strength of X and the p*K*_a_ of its free ligand form as a proxy; for instance, [Ru­(SO_2_)­(NH_3_)_4_(isonicotinamide)]­[tosylate]_2_ (PCE = 39.8%, p*K*_a_ (isonicotinamide) = 10.61) shows a higher PCE than [Ru­(SO_2_)­(NH_3_)_4_(isonicotinic acid)]­[tosylate]_2_ (PCE = 35.7%, p*K*_a_ (isonicotinic acid) = 4.96).^23^ However, such pairwise comparisons conflate σ-donor strength with covarying electronic and steric properties of X, and this correlation does not extend across the family: e.g., [Ru­(SO_2_)­(NH_3_)_4_(3-phenylpyridine)]­Cl_2_·H_2_O and [Ru­(SO_2_)­(NH_3_)_4_(pyridine)]­Cl_2_·H_2_O achieve PCEs of 52(3)% and 57.7(11)%, respectively, despite the ligand p*K*_a_ of 3-phenylpyridine (p*K*_a_ = 4.73) being less than that of pyridine (p*K*_a_ = 5.12).^10,27^ Thus, the σ-donor ability of these Ru-based complexes is at best a partial descriptor of their photoresponsive properties. Geometric descriptors such as the Voronoi–Dirichlet polyhedral (VDP) volume^10^ have likewise shown predictive power only within restricted subsets of this family of complexes. Therefore, a unifying account of these structure–property relationships requires a direct interrogation of their electronic structure rather than wholly relying upon empirically determined molecular descriptors.

In this work, we investigate the photoisomerization behaviors of a series of these Ru-based complexes which feature a diverse range of *trans*-ligands and counterions (X = pyridine, 3-F-pyridine, 3-Cl-pyridine, 3-Br-pyridine, 3-I-pyridine, 4-Cl-pyridine, 4-Br-pyridine, H_2_O; Y = tosylate, Cl_2_·H_2_O).^11,22,25–29^ Our aim is to elucidate how this *trans*-ligand identity influences photoisomerization behavior and PCE. These 11 compounds were chosen not only because they share similar *trans*-ligands and counterions, X and Y, but also because their light-induced crystal structures have been characterized experimentally. By combining electronic-structure analysis with response charge-density calculations, we compare the orbital interactions that govern [Ru]-S bond strength and quantify light-induced charge transfer across these molecular crystal structures. Furthermore, we assess the predictive potential of the *d*-band center as a molecular-scale design descriptor for PCE and explain why the *d*-band center exhibits a quasi-linear relationship with experimentally measured PCEs. This integrated approach provides new mechanistic insight into key structure–property relationships within the [Ru­(SO_2_)­(NH_3_)_4_X]Y family, and offers guiding principles for the rational design of high-efficiency, multifunctional photoactive materials.

All spin-polarized first-principles calculations were performed using density functional theory (DFT) as implemented in the *Quantum ESPRESSO* (QE) package, version 6.3.^36^ A total of 11 crystal structures were investigated, although the results from four representative [Ru­(SO_2_)­(NH_3_)_4_X]­[tosylate]_2_ complexes (X = H_2_O (**1**), 3-F-pyridine (**2**), 3-Cl-pyridine (**3**), and 3-Br-pyridine (**4**)) are presented in the main body of this paper for the purpose of clarity; results on the other complexes are provided in the Supporting Information (SI). Three considerations guide our choice of complexes to present herein. First, the four complexes span the full range of experimental PCEs reported for the chemical family, from 11.1% in **1** to 100% in **4**, so that the mechanistic comparison covers both the low- and high-PCE limits. Second, they feature two categories of *trans*-ligand variation: nonpyridine versus pyridine (**1** versus **2–4**), and a halogen series within the 3-halopyridine ligand identity (**2** versus **3** versus **4**). Third, the projected density of states (PDOS) profiles computed for all 11 complexes (Figures 2, 4, and S1–S9) show qualitatively consistent dark-state electronic structures across the series, indicating that the mechanistic conclusions drawn from these four representatives generalize to the full set.

Initial atomic coordinate geometries for each complex were taken from previously reported crystal structures that have been determined via single-crystal X-ray diffraction, for which the associated synthesis and characterization of these complexes are also described.^10,11,22–35^ In periodic calculations, electronic states are sampled at wavevectors (k-points) within the Brillouin zone, the unit cell of reciprocal space, whose center (k = 0) is the Γ point. The *turbo_davidson* and *turbo_lanczos* modules used for the excited-state calculations support Γ-point sampling only;^37,38^ the ground-state calculations were therefore also performed at the Γ point, so that both sets of calculations share the same Kohn–Sham states. This restriction is justified for the present molecular crystals: their lattice parameters (e.g., **a** = 6.73 Å, **b** = 12.72 Å, **c** = 13.37 Å for complex **1**) correspondingly reduce the extent of the Brillouin zone in reciprocal space, and the weak intermolecular interactions result in negligible band dispersion, as evidenced by the narrow PDOS features discussed below. The Perdew–Burke–Ernzerhof exchange-correlation functional for solids (PBEsol) was employed within the generalized gradient approximation (GGA).^39,40^ Van der Waals interactions were accounted for using the Grimme D3 dispersion correction.^41–43^ The kinetic energy cut-offs for the wave function (E_Ψ_) and the charge density (E_ρ_) were set to E_Ψ_ = 280 Ry and E_ρ_ = 4 × E_Ψ_ = 1120 Ry, respectively. All DFT calculations were converged with respect to Γ-point sampling and plane-wave cutoff until the total energy change was below 1 × 10^–10^ Ry. The optimized geometries were subsequently used as input for time-dependent density functional theory (TDDFT) calculations to determine the optical properties of the complexes. Photoexcited-state calculations and UV–vis spectral simulations were carried out using the *turbo_davidson* and *turbo_lanczos* modules within QE.^37,38^ For the *turbo_lanczos* calculations, 13,000 Lanczos iterations were performed to obtain the full polarizability tensors and absorption coefficients. For the *turbo_davidson* calculations, 25 eigenvalues were computed with a convergence threshold of 1 × 10^–4^ a.u.

Before analyzing photoexcited-state changes in the [Ru­(SO_2_)­(NH_3_)_4_X]­Y complexes using TDDFT, we first established the dark-state electronic structure and bonding picture of each complex. For **1**, the PDOS for Ru with S (from the photoactive SO_2_ ligand) and O (from the *trans*-ligand, H_2_O) is shown in Figure 2(a) and 2(b). The PDOS profile exhibits two characteristic features of these Ru-based crystal structures: (i) narrow, spiky peaks near the valence-band maximum (VBM) and conduction-band minimum (CBM), reflecting weak intermolecular coupling and typical molecular-like discrete states; and (ii) broader manifolds arising from ligand *p* states and Ru *d* states that exhibit finite band dispersion. For such compounds, it is convenient, by analogy with isolated molecules, to refer to the band-edge states as HOMO- and LUMO-derived levels.

**Figure 2:**
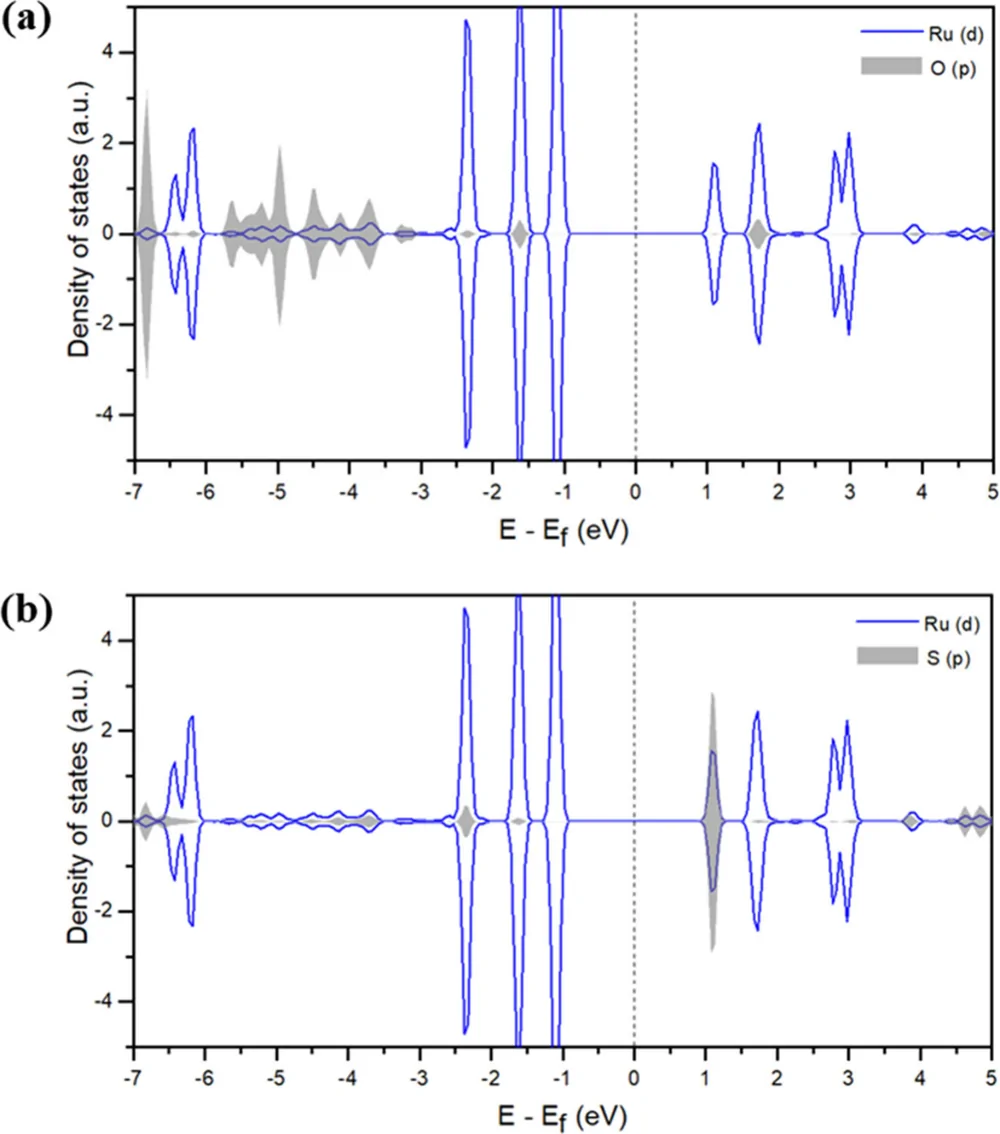
Projected density of states (PDOS) for (a) Ru and O (from the *trans*-ligand H_2_O), and (b) Ru and S (from the photoactive SO_2_ ligand) in the dark-state crystal structure of [Ru­(SO_2_)­(NH_3_)_4_(H_2_O)]­[tosylate]_2_ (**1**). PDOS contributions from ligand *p*-states are shown in gray; those of Ru *d*-states are given in blue. The dashed line indicates the Fermi level.

From these PDOS profiles, the bonding interactions between Ru and S (from the photoactive SO_2_ ligand), as well as between Ru and O (from the *trans*-ligand, H_2_O) in **1** can be identified. For the [Ru]-O coordinative bond, two dominant interactions are evident (Figure 2 and Figure 3): a π-type interaction between the Ru *d*_*yz*_ orbital and the O *p*_*y*_ orbital at −1.60 eV, and a σ-type interaction between the Ru *d*_z^2^_ orbital and the O *p*_*z*_ orbital at −3.20 eV. For the [Ru]-S bond within the photoactive SO_2_ ligand, a single pronounced π-type interaction between the S *p*_*y*_ orbital and the Ru *d*_*yz*_ orbital is observed at −2.32 eV. The HOMO-derived state is primarily localized on the Ru *d*_*xy*_ orbital, whereas the LUMO-derived state is distributed over the SO_2_ ligand and the Ru center, with negligible contribution from the O atom of the *trans*-ligand.

**Figure 3:**
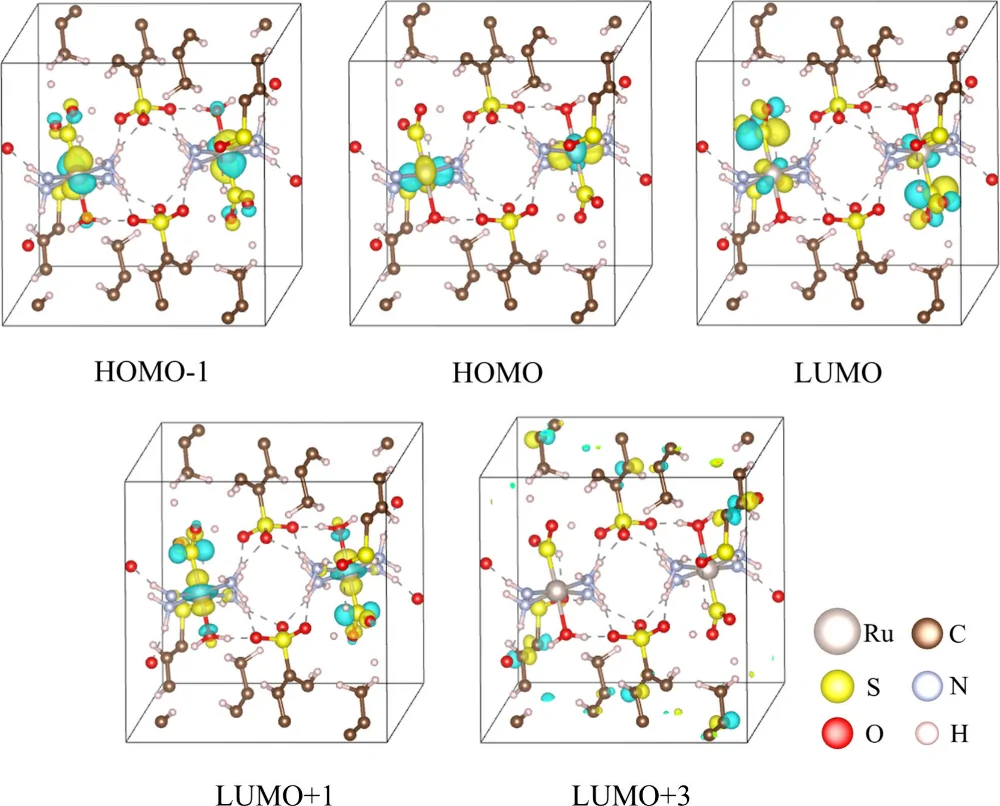
Isosurface (0.003 a.u.) of the frontier orbitals of [Ru­(SO_2_)­(NH_3_)_4_(H_2_O)] [tosylate]_2_ (**1**); yellow and blue regions represent the two different phases of the wave function.

The PDOS profiles of **2**–**4** bearing a 3-halopyridyl-based *trans*-ligand were also examined to identify differences from the electronic structure of **1** (bearing the *trans*-ligand, H_2_O). The bonding characteristics of **2**–**4** were found to be largely similar to each other, so only the electronic structure of **2** is shown in Figure 4(a) and 4(b) for brevity; those of **3** and **4** are provided in Figures S1 and S2 of the SI. Visualizations of the frontier orbitals for **2**–**4** are shown in Figures S11–S13, respectively. For the Ru center and the N atom of the 3-halopyridyl-based *trans*-ligand in **2**–**4**, two prominent [Ru]-N bonding interactions are evident: a π-type interaction between Ru *d*_*yz*_ and N *p*_*y*_ (−3.75 eV for **2**, see Figure 4(a); −3.62 eV for **3**, see Figure S1­(a); −3.71 eV for **4**, see Figure S2­(a)) and a σ-type interaction between Ru *d*_*z* ^2^_ and N *p*_*z*_ (−3.70 eV for **2**, see Figure 4(a); −3.79 eV for **3**, see Figure S1­(a); −3.82 eV for **4**, see Figure S2­(a)). For the [Ru]-S coordinative bond involving the photoactive SO_2_ ligand, only a single π-type interaction between Ru *d*_*yz*_ and S *p*_*y*_ is observed (−2.05 eV for **2**, see Figure 4(b); −2.11 eV for **3**, see Figure S1­(b); −2.15 eV for **4**, see Figure S2­(b)). A notable difference from **1** is that the LUMO-derived states of **2**–**4** show significant contributions from the photoactive SO_2_ ligand, the Ru center, and the pyridyl nitrogen (cf. Figures S11–S13). This extended LUMO delocalization implies that the [Ru]-N_pyridyl_ bond in **2**–**4** is more susceptible to electronic reorganization upon photoexcitation than the dark-state [Ru]-O bond in **1**. This observation is consistent with experimental evidence, in which the PCEs of **2**–**4** are substantially higher than that of **1**.^22,25,26,28,34^

**Figure 4:**
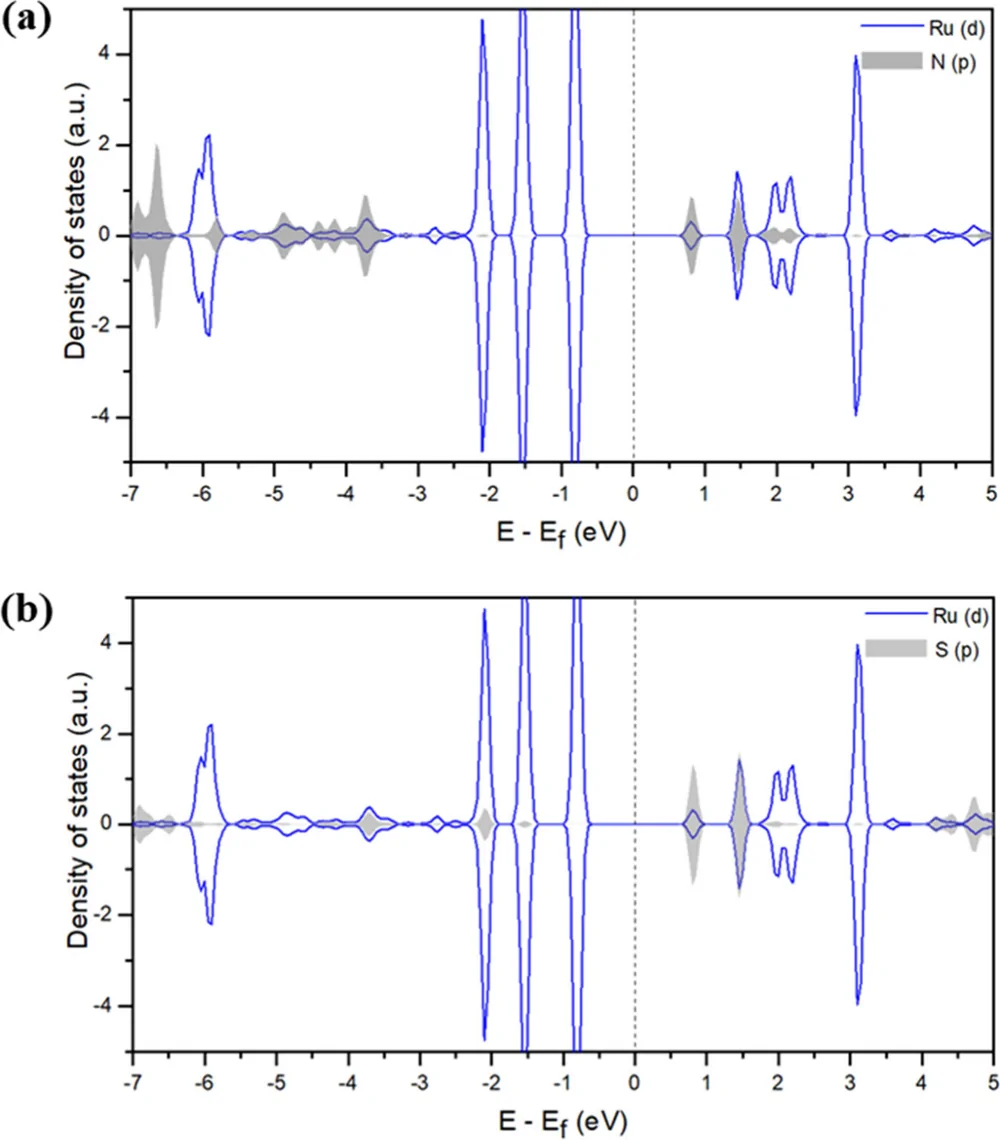
Projected density of states (PDOS) for (a) Ru and N (from the *trans*-ligand 3-F-pyridine), as well as (b) Ru and S (from the photoactive SO_2_ ligand) in [Ru­(SO_2_)­(NH_3_)_4_(3-F-pyridine)]­[tosylate]_2_ (**2**). PDOS contributions from ligand *p*-states are shown in gray; those of Ru *d*-states are given in blue. The dashed line indicates the Fermi level.

TDDFT calculations were then carried out to compute the optical absorption spectra of these complexes. Such information can elucidate how the electronic structural characteristics of the Ru coordinative center in these complexes change upon photoexcitation, and provide further insights for the molecular design of [Ru­(SO_2_)­(NH_3_)_4_X]Y photoresponsive materials. Before drawing conclusions from the computed results, it is essential to assess the agreement between theory and experiment; the latter had previously been acquired using a custom experimental setup for single-crystal optical absorption spectroscopy.^32^ As an example, the calculated optical absorption spectrum of **1** is compared with its experimentally measured counterpart in Figure S10. The theoretical spectrum reproduces the two main absorption features that were observed experimentally, one in the range of 450–500 nm and another between 550 and 700 nm, although the experimental peaks are considerably broader. The broadening is reasonable because the theoretical spectrum is obtained from a single dark-state-optimized crystallographic geometry and captures neither the Franck–Condon vibrational envelope associated with each electronic transition nor the thermal sampling of nuclear coordinates at the experimental temperature (∼100 K), both of which add broadening to the experimental absorption profile.

Discrepancies in peak wavelengths between the computed and experimental spectra of **1** (Figure S10) are also evident. In plane-wave TDDFT with semilocal GGA functionals, computed excitation energies are shifted by self-interaction error and by the absence of long-range exchange, particularly for charge-transfer transitions.^44–47^ These offsets can be reduced by hybrid functionals,^38,48^ but at a cost that is demanding for the unit cells studied here. The single-geometry treatment also omits vibronic and thermal broadening.^49^ We therefore compare only transitions of similar character across structurally similar complexes that were treated using the same level of theory, assuming that all are equally affected by these approximations.

Having confirmed the overall consistency between experiment and theory, we analyzed the main optical transitions in detail. The transition energies, wavelengths, oscillator strengths, and dominant orbital contributions are summarized in Table 1, and the corresponding molecular orbitals are illustrated in Figure 3. For **1**, the lowest-energy absorption is dominated by the HOMO → LUMO excitation. The HOMO is primarily metal-centered (Ru *d*_*xy*_), while the LUMO carries a significant contribution from the *p*_*y*_ orbital of the SO_2_ ligand, so that this lowest-energy transition exhibits a predominantly metal-to-ligand charge-transfer (MLCT) character. The HOMO–1→LUMO transition at 458 nm (f = 0.13) originates from an orbital of Ru *d*_*xz*_ and SO_2_ *p*_*z*_ character, which carries no [Ru]-S *d*_*yz*_-*p*_*y*_ bonding character.

**Table 1:** Transition Energies, Wavelengths, Oscillator Strengths, and Electron-Hole Components for [Ru­(SO_2_)­(NH_3_)_4_(H_2_O)]­[tosylate]_2_ (**1**) in the Visible Range (400−800 nm) of Absorption^a^

			Major contributions	
Energy (eV)	Wavelength (nm)	Oscillator strength		
2.33	532	0.19	HOMO→LUMO	98.89%
2.71	458	0.13	HOMO–1→LUMO	99.80%
2.79	444	0.11	HOMO→LUMO+1	95.82%
3.00	413	0.18	HOMO→LUMO+1	73.04%
			HOMO→LUMO+3	19.31%

a Only contributions ≥5% are reported.

Analysis of the lowest-energy transition in **1** also explains why approximately 11.1% of the [Ru]-S-bound dark state converts to the side-bound [Ru]-(OS)O state (MS2) upon photoexcitation. As shown in Figure 3, the LUMO of **1** exhibits π*-antibonding character arising from the out-of-phase combination of the S *p*_*y*_ and Ru *d*_*yz*_ orbitals. The HOMO → LUMO excitation populates this antibonding state, thereby weakening the [Ru]-S bond and lowering the energy barrier for SO_2_ linkage photoisomerization. At the same time, Table 1 shows that the oscillator strengths for all identified photoexcited transitions in **1** are small (≤0.2 a.u.), which explains why photoisomerization can still occur in **1**, albeit with a modest PCE: although weak absorption limits the fraction of complexes that are photoexcited within a crystal of **1**, those that are photoexcited experience a destabilized [Ru]-S bond at a level that is sufficient to undergo photoisomerization.

To clarify the differences in photoisomerization behavior among **1**–**4**, the detailed optical absorption data and orbital assignments for **2**–**4** are provided in Tables S1–S3 and Figures S11–S13. The lowest-energy transitions in **2**–**4** occur at lower energies than in **1** (1.60 eV, 1.63 and 1.64 eV for **2**, **3**, **4**, respectively, versus 2.33 eV for **1**); this is consistent with the modified dark-state electronic structures of **2**–**4** identified above. Beyond this red shift, two further differences are quantitatively important. First, the cumulative oscillator strength of the transitions in the visible range of the electromagnetic spectrum increases markedly on passing through the chemical series from **1** (all f ≤ 0.19) to **2** and **3** (several transitions reach f = 0.40–0.57) to **4**, where the transition at 429 nm attains the largest oscillator strength, f = 1.36. Second, in addition to the HOMO → LUMO MLCT transition which is common to the entire series, **2**–**4** display a secondary class of transitions in which a [Ru]-S (*d*_*yz*_-*p*_*y*_) bonding orbital is depopulated while the corresponding antibonding LUMO is simultaneously populated; thus, producing a concurrent depletion of a bonding state and the filling of an antibonding state that we refer to below as the dual-weakening channel.

Our theoretical results provide strong evidence that the photoinduced reorganization of the Ru-centered electronic structure directly influences the [Ru]-S and [Ru]-X bonds, thereby modulating the PCE. For **1**, both the [Ru]-S and [Ru]-O_H2O_ bonds elongate upon photoexcitation, as the corresponding antibonding orbitals, [Ru]-S (*d*_*yz*_-*p*_*y*_) and [Ru]-O_H2O_ (*d*_*z* ^2^_-*p*_*z*_), are populated via the HOMO → LUMO (532 nm) and HOMO → LUMO+1 (444 nm) transitions. Similar behavior is observed in **2**–**4**, where the [Ru]-S and [Ru]-N_pyridyl_ bond-length changes are more pronounced than the corresponding changes in **1**. Among the 3-halopyridyl complexes, **4** exhibits the largest photostructural distortion; this trend is consistent with a substantial population of antibonding orbitals for both [Ru]-S (*d*_*yz*_-*p*_*y*_) and [Ru]-N_pyridyl_ (*d*_*z* ^2^_-*p*_*z*_) coordination, as documented in Table S3 and Figure S13: LUMO+1 and LUMO+3 of **4** contain substantial metal-centered *d*-antibonding contributions and display strong π*- and σ*-antibonding character, respectively, and their population upon photoexcitation further weakens the [Ru]-S and [Ru]-N_pyridyl_ coordinative bonding.

A particularly informative feature of **4** is its exceptionally strong absorption at 429 nm (f = 1.36), which is assigned primarily to the HOMO–3 → LUMO transition (82.42%, with a minor HOMO–2 → LUMO contribution of 15.43%; Table S3). The HOMO–3 orbital of **4** (Figure S13) carries *d*_*yz*_-*p*_*y*_ π-bonding character that is localized on the [Ru]-S bond, so that the photoexcitation of **4** simultaneously depopulates the bonding orbital and populates the antibonding LUMO. This dual-weakening channel is not unique to **4**: analogous HOMO–2 → LUMO transitions are found in **2** (426 nm, f = 0.55) and **3** (425 nm, f = 0.47). What distinguishes **4** is the oscillator strength of its dual-weakening transition, which is more than twice that of **2** and nearly three times that of **3**. Under irradiation within its optical absorption band, the dual-weakening channel is therefore populated far more efficiently in **4**, which is a major contributing factor to the large [Ru]-S elongation and the superior PCE (100%) that is experimentally observed for this complex.^26^

The subtle differences in PCE among **2**–**4** were next examined by analyzing their electronic response at selected photoexcitation wavelengths, as shown in Figure 5. This figure presents the imaginary (absorptive) component of the charge-density response, *F*(**r**, λ), induced by the incident electric field for the three 3-halopyridyl-ligated Ru-based structures at 426 nm (**2**), 425 nm (**3**), and 429 nm (**4**), respectively. These wavelengths were chosen because they correspond to transitions with dominant contributions from the *d*_*yz*_-*p*_*y*_ orbital, which mediates the principal [Ru]-S π-interaction identified above. Comparing *F*(**r**, λ) at these nearly identical wavelengths therefore provides a direct measure of the photoinduced perturbation to the [Ru]-S bond across these three complexes under equivalent photoexcitation conditions.

**Figure 5:**
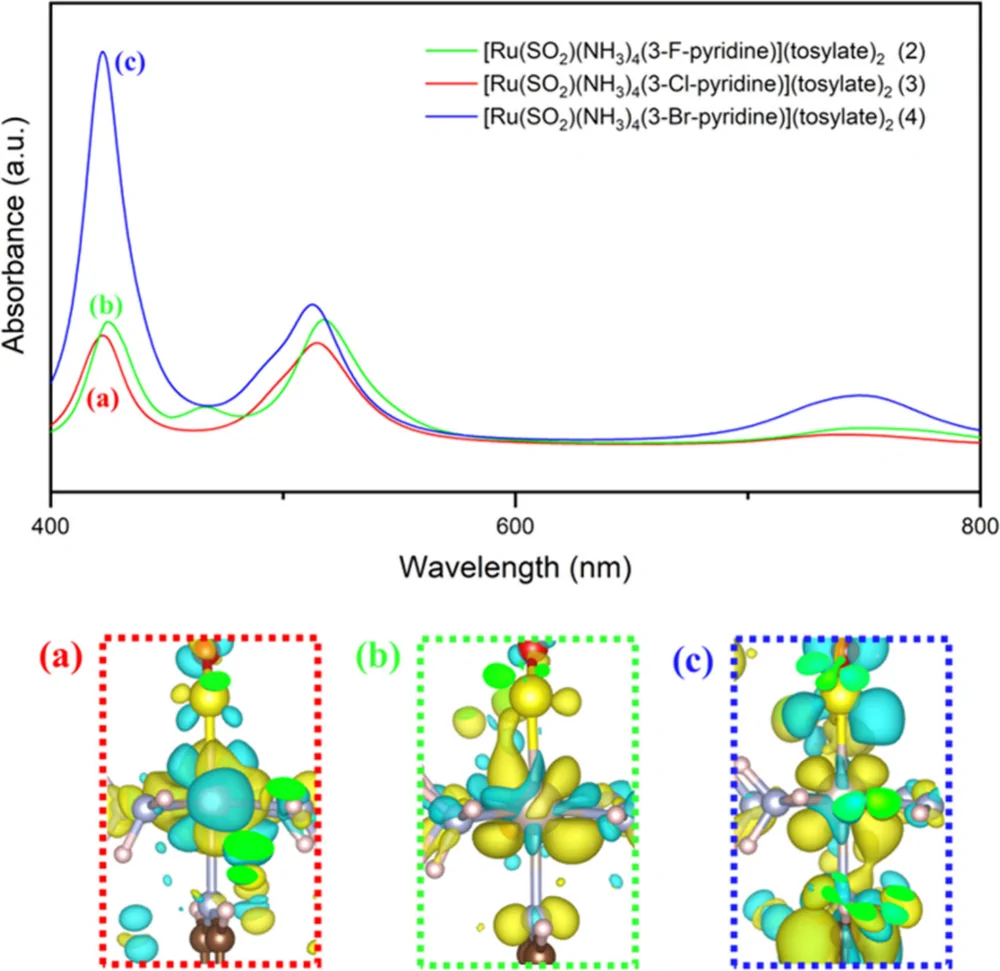
Absorption spectra (top) and isosurface plots (isosurface = 0.0005 a.u.) of the imaginary component of the charge-density response *F*(**r**, λ) (bottom) for (a) [Ru­(SO_2_)­(NH_3_)_4_(3-Cl-pyridine)]­[tosylate]_2_ (**3**) at 425 nm, (b) [Ru­(SO_2_)­(NH_3_)_4_(3-F-pyridine)]­[tosylate]_2_ (**2**) at 426 nm, and (c) [Ru­(SO_2_)­(NH_3_)_4_(3-Br-pyridine)]­[tosylate]_2_ (**4**) at 429 nm. Yellow and blue regions represent charge accumulation and depletion, respectively; green regions are cross sections of the isosurfaces where they are cut by the plot boundary and carry no physical meaning.

Figure 5 shows a progressively stronger charge-density response from spectra (**a**)–(**c**) in the region between the Ru center and the SO_2_ ligand upon photoexcitation, following the order **3** < **2** < **4**. The increasing magnitude of *F*(**r**, λ) along this series reflects a greater redistribution of electron density away from the [Ru]-S bonding region upon photoexcitation, which is consistent with an increasing degree of [Ru]-S bond weakening; this finding mirrors the experimentally observed PCE trend. While our analysis of the PDOS and electronic transitions describes the energetics and symmetry of the individual photoexcitations, *F*(**r**, λ) provides a real-space visualization of these transitions, showing where the photoinduced charge redistribution occurs. At the common isovalue used, the response in **4** (Figure 5(c)) also extends beyond the immediate [Ru]-S bond into the surrounding SO_2_ ligand, as expected from its larger oscillator strength.

The detailed analysis of the electronic structures of all 11 Ru-based complexes in this study before and after photoexcitation highlights the significant involvement of Ru *d*-electrons in the SO_2_ photoisomerization process. In particular, the TDDFT results reveal that the key transitions governing [Ru]-S bond weakening, and hence the PCE, involve photoexcitations between Ru *d*-derived bonding and antibonding states. This behavior is reminiscent of transition-metal electrocatalysis, most notably the oxygen evolution reaction (OER), where the position of the *d*-band center correlates linearly with the catalytic overpotential.^50,51^ Although the physical context differs, the underlying principle is analogous: the position of the Ru *d*-band center governs the energy of the metal–ligand antibonding states and thereby controls the ease with which these states can be populated. Motivated by this analogy, we evaluated the Ru *d*-band center as a molecular-scale descriptor for predicting the PCE of [Ru­(SO_2_)­(NH_3_)_4_X]Y complexes. The form that this descriptor takes follows the mechanism established above. Within the *d*-band picture, the strength of the [Ru]-S interaction is governed both by the position of the Ru *d* states and by their coupling to the S *p*_*y*_ orbital; the latter is set by the electronic character of the *trans*-ligand, which competes for the same Ru *d* orbitals along the S–Ru–X axis. Complexes sharing a *trans*-ligand class therefore share a coupling regime, and within such a class, the PCE varies with the Ru *d*-band center. Changing the class of *trans*-ligand changes both the coupling and the competing [Ru]-X interaction and so displaces the correlation. The descriptor is thus two-tiered: the class of *trans*-ligand determines which trend a complex follows, and the *d*-band center captures the direction of that trend.

The 11 structures comprise two such classes. Ten bear a pyridyl *trans*-ligand whose low-lying π* manifold mixes with the Ru *d* states and so enables the dual-weakening channel identified above for **2**–**4**; these define the trend in Figure 6(a), within which the tosylate and Cl_2_·H_2_O salts are distinguished into two subclasses by the counterion identity. The second class exclusively comprise **1** whose aqua *trans*-ligand is a pure σ-donor with no π* manifold, so **1** cannot access that channel. It constitutes the second class, and its displacement from the pyridyl trend marks a distinct trend rather than an anomaly, one which the single structure available here is insufficient to define; it is therefore excluded from the regression.

**Figure 6:**
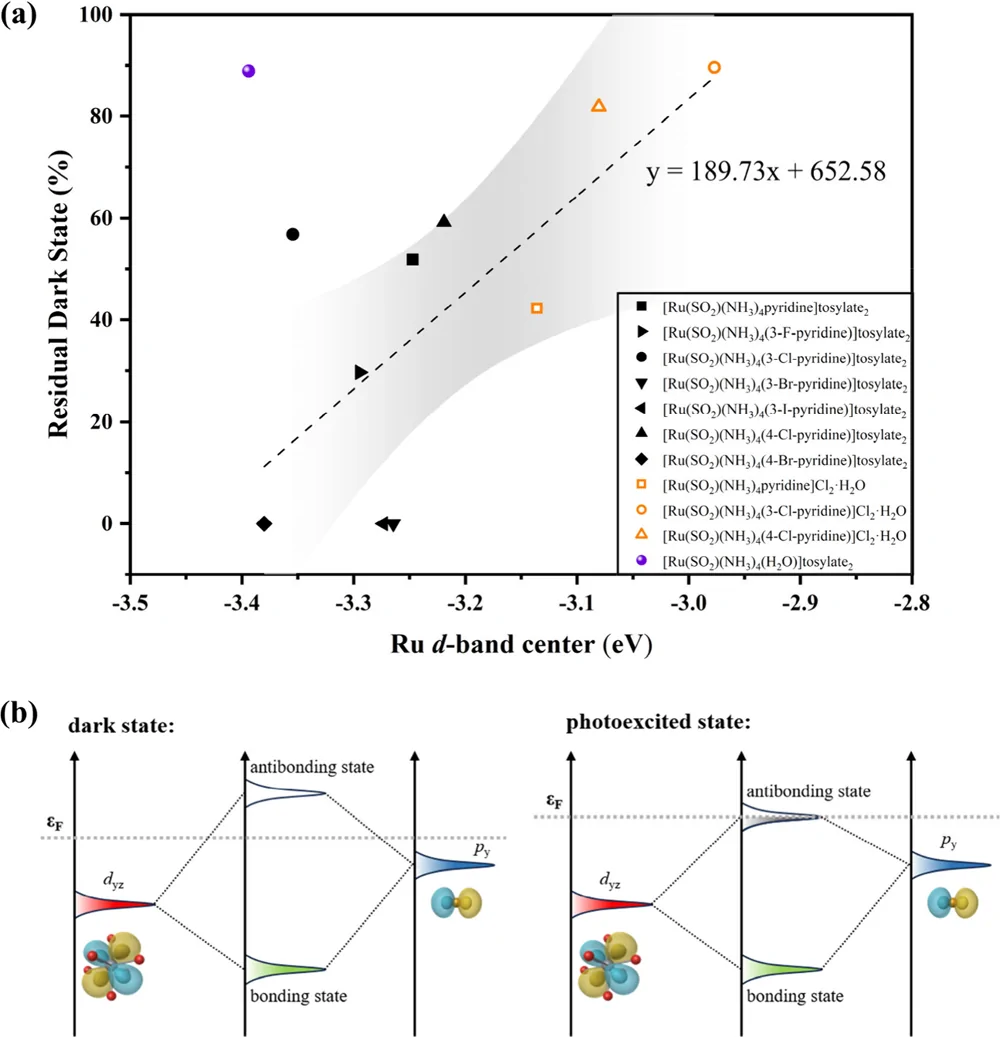
(a) Percentage of residual dark state (S-bound η^1^-SO_2_ configuration) remaining after irradiation, plotted against the calculated Ru *d*-band center for the 11 [Ru­(SO_2_)­(NH_3_)_4_X]Y complexes examined (X = pyridine, 3-F-pyridine, 3-Cl-pyridine, 3-Br-pyridine, 3-I-pyridine, 4-Cl-pyridine, 4-Br-pyridine, H_2_O; Y = tosylate, Cl_2_·H_2_O). Black filled symbols denote pyridyl tosylate salts, orange open symbols denote Cl_2_·H_2_O salts, and the purple symbol denotes the aqua complex [Ru­(SO_2_)­(NH_3_)_4_(H_2_O)]­[tosylate]_2_ (**1**), whose *trans*-ligand places it in a second class; it is excluded from the fit. The dashed black line is a least-squares fit to the ten pyridyl-ligated complexes, and the gray shaded region is its 95% confidence band. Calculated *d*-band centers, literature-reported photoconversion efficiencies (PCEs) and irradiation wavelengths for all 11 complexes are listed in Table S4. (b) Schematic of the [Ru]-S interaction formed between the Ru *d*_*yz*_ and S *p*_*y*_ orbitals in the dark state (left) and the photoexcited state (right). Orbital isosurfaces of *d*_*yz*_ and *p*_*y*_ are shown alongside the corresponding projected densities of states, and the dashed line marks the Fermi level *(ε*_*F*_ *)*. In the dark state the [Ru]-S antibonding combination lies above *ε*_*F*_ and is unoccupied; photoexcitation populates this state, reducing the [Ru]-S bond order.

The Ru *d*-band center (*ε*_*c*,*F*_) is calculated as the first moment of the Ru *d*-projected density of states, following Hammer and Nørskov,^52,53^ with the integration performed between ε_1_ = −20 eV and ε_2_ = +10 eV relative to the Fermi level, a window chosen to encompass the entire Ru *d* manifold:

$$\varepsilon_{c,F}=\frac{\int_{\varepsilon 1}^{\varepsilon 2}E\times {\mathrm{PDOS}}_{F}\left(E\right)dE}{\int_{\varepsilon 1}^{\varepsilon 2}{\mathrm{PDOS}}_{F}\left(E\right)dE}$$

where PDOS_*F*_(*E*) is the projection of the density of states onto the Ru *d* orbitals at a certain energy level (*E*). Figure 6(a) presents the relationship between the calculated *d*-band centers for all structures studied here and their experimentally measured PCE values.

Figure 6(a) shows the calculated Ru *d*-band centers plotted against the residual dark-state fraction for all 11 structures. A Ru *d*-band center lying closer to the Fermi level corresponds to a larger residual dark-state fraction, and hence a lower PCE. A least-squares fit over the ten pyridyl-ligated complexes gives y = 189.73x + 652.58, where y is the residual dark-state fraction (%) and x is the Ru *d*-band center (eV). The scatter about this line is consistent with the multifactorial nature of the photoisomerization process. The *d*-band center is therefore best regarded as a screening descriptor that captures the direction of the trend, rather than as a quantitative predictor of the PCE. It does not resolve the finer ordering among **2**–**4** (**3 < 2 < 4**), which is instead reflected in the oscillator strength of the dual-weakening transition discussed above, visualized in real space by the charge-density response (Figure 5), although other factors, such as the steric constraints of the photoreactive cavity, may also contribute to the observed PCE trend. Note that our regression process was not divided into two least-squares fitting processes, one for each subclass of the two salt families, given the limited amount of data involved. To this end, our rationale is 2-fold. First, a regression on the three Cl_2_·H_2_O salts retains a single residual degree of freedom, for which the critical t value is 12.7; the 95% confidence interval on the slope therefore spans ±12.7 standard errors. A rank correlation is no more informative: three points admit only 3! = 6 rankings, so even a perfectly monotonic ranking gives a one-sided p of 1/6, well above the 5% significance level. Second, Table S4 shows that the counterion is confounded with the irradiation protocol: all three Cl_2_·H_2_O salts were characterized under broadband 425–650 nm irradiation, whereas the seven pyridyl-based tosylate salts include four measured at 505 nm and three under broadband irradiation. Any difference between the two families could therefore not be attributed to the counterion.

The physical basis for the observed PCE trend involves two stages, illustrated schematically in Figure 6(b). In the dark state, the [Ru]-S interaction is dominated by π-type overlap between the filled Ru *d*_*yz*_ orbital and the vacant S *p*_*y*_ component of the SO_2_ π* manifold.^52,53^ The corresponding [Ru]-S antibonding combination is the LUMO-derived state and is unoccupied, so it contributes no repulsive term.^54^ Within the *d*-band model, the interaction strength is instead governed by the stabilization of the occupied bonding combination, which scales inversely with the energy separation between the Ru *d* states and the S *p*_*y*_ states. As the Ru *d*-band center shifts upward toward the Fermi level this separation decreases, *d*_*yz*_-*p*_*y*_ hybridization strengthens, and Ru→SO_2_ back-donation is enhanced.^55^ The dark-state Ru *d*-band center therefore sets the intrinsic [Ru]-S bond strength across the series.

Upon photoexcitation, this antibonding state is populated. The TDDFT analysis above shows that the lowest-energy MLCT transition transfers density from Ru into the [Ru]-S antibonding LUMO in all four complexes examined in detail, while **2**–**4** access an additional channel in which a [Ru]-S bonding orbital is simultaneously depopulated. Both processes reduce the [Ru]-S bond order and lower the barrier to SO_2_ linkage photoisomerization. Since this photoinduced weakening acts on a bond whose initial strength is set by the Ru *d*-band center, complexes beginning from a weaker [Ru]-S bond require less destabilization to reach the photoisomerization threshold, and a larger fraction of the crystal converts to the metastable states. Predicting photoexcited-state behavior from dark-state electronic structure in this way is well established for d^6^ ruthenium complexes: the dark-state ligand-field strength determines the energy of the metal-centered excited state and hence the photosubstitution efficiency,^56^ and in the closely related *trans*-[Ru­(NO)­(NH_3_)_4_X]^2+^ complexes the NO linkage photoisomerization barrier correlates linearly with a dark-state orbital energy.^57^

Two complexes lie away from the fitted trend shown in Figure 6(a). The first is [Ru­(SO_2_)­(NH_3_)_4_(H_2_O)]­[tosylate]_2_ (**1**), whose aqua *trans*-ligand places it in a separate class, as discussed above. The second is [Ru­(SO_2_)­(NH_3_)_4_(3-Cl-pyridine)]­[tosylate]_2_ (**3**), which shows the largest deviation among the pyridyl-ligated complexes. The PCEs of the ten pyridyl-ligated structures were obtained under two irradiation protocols, broadband 425–650 nm and monochromatic 505 nm (Table S4),^10,11,22–33,35^ with that of **3** determined under the former.^25^ Since PCEs obtained under different irradiation protocols are not strictly comparable, the linearity of the trend cannot be reliably assessed from the present data, and the deviation of **3** is therefore not interpreted further. As such, our results highlight the need to determine the PCE of **3** under 505 nm light irradiation, which would allow the linearity of the partially established trend to be fully assessed for [Ru­(SO_2_)­(NH_3_)_4_X]Y complexes where X is a 3-halopyridyl *trans*-ligand; accordingly, this is the subject of ongoing work.

In conclusion, we have systematically investigated the dark-state and photoexcited-state electronic structures of [Ru­(SO_2_)­(NH_3_)_4_X]Y complexes in their single-crystal form and elucidated how the identity of the *trans*-ligand X modulates their SO_2_ linkage photoisomerization behavior. For clarity, four representative crystal structures of [Ru­(SO_2_)­(NH_3_)_4_(H_2_O)]­[tosylate]_2_ (**1**), [Ru­(SO_2_)­(NH_3_)_4_(3-F-pyridine)]­[tosylate]_2_ (**2**), [Ru­(SO_2_)­(NH_3_)_4_(3-Cl-pyridine)] [tosylate]_2_ (**3**), and [Ru­(SO_2_)­(NH_3_)_4_(3-Br-pyridine)]­[tosylate]_2_ (**4**), were selected to illustrate the bonding changes upon photoexcitation. In the dark state, the S atom of the photoactive SO_2_ ligand and the *trans*-ligand coordinate to Ru in a similar fashion: two bond types, π-type (*d*_*yz*_-*p*_*y*_) and σ-type (*d*_*z* ^2^_-*p*_*z*_), are formed between Ru and the *trans*-ligand, while only a π-type (*d*_*yz*_-*p*_*y*_) interaction is found between Ru and the S atom of the SO_2_ ligand. However, the electronic-structure dynamics of these complexes diverge upon photoexcitation. In **1**, photoexcitation populates the antibonding *d*_*yz*_-*p*_*y*_ orbital, weakening the [Ru]-S bond and lowering the barrier for SO_2_ linkage photoisomerization. In **2–4**, photoexcitation not only populates this antibonding orbital but also depopulates its bonding counterpart, leading to a more pronounced weakening of the [Ru]-S bond and, consequently, higher PCE than in **1**.

The finer ordering among **2–4** cannot be explained by σ-donor strength alone, since 3-Cl-pyridine and 3-Br-pyridine share an identical free-ligand p*K*_a_ of 2.84. Instead, three concurrent effects of the *trans*-ligand, X, on the PCE can be identified: (i) tuning of the optical gap and hence the spectral overlap with the irradiation source, (ii) redistribution of oscillator strength across the visible range of optical absorption, and (iii) control over the efficiency of the bonding/antibonding channels involved in the photoexcitation. Together, these factors rationalize the experimentally observed nonmonotonic PCE ordering **4** > **2** > **3** > **1**. Calculations of the charge-density response in the wavelength range 425–429 nm visualize the corresponding light-induced charge redistribution in real space; its magnitude increases in the order **3** < **2** < **4**, in line with the oscillator strengths of the dual-weakening transitions.

Given the central role of Ru *d*-states in the photoexcitation process, we evaluated the *d*-band center as a single-parameter descriptor for the PCE in single crystals of [Ru­(SO_2_)­(NH_3_)_4_X]Y complexes. While the *d*-band center is a useful screening descriptor, it should be remembered that the photoisomerization process is multifactorial, with additional influences from the steric photoreactive cavity volume,^25^ photoexcitation wavelength,^58^ and temperature of the single crystal.^22^ Extending the present analysis to [Ru]-SO_2_ bonding in the η^1^-OSO and η^2^-(OS)O metastable states would further clarify the photoisomerization pathway. Looking ahead, as more extensive experimental data sets of crystal structures become available from photocrystallography, robust multiparameter descriptors could be developed via statistical-learning approaches to account for these factors and to facilitate the rational design of photoresponsive single-crystal materials with high PCE for quantum photosensing applications.

## Supplementary Material





## Notes

Crystallographic data reformatted for electronic structure calculations reported in this study (ZIP)
PDOS for Ru and N (from *trans*-ligand 3-Cl-pyridine), as well as Ru and S (from the photoactive SO_2_ ligand) in [Ru­(SO_2_)­(NH_3_)_4_(3-Cl-pyridine)]­[tosylate]_2_; PDOS for Ru and N (from *trans*-ligand 3-Br-pyridine), as well as Ru and S (from the photoactive SO_2_ ligand) in [Ru­(SO_2_)­(NH_3_)_4_(3-Br-pyridine)]­[tosylate]_2_; PDOS for (a) Ru and N (from *trans*-ligand pyridine), as well as (b) Ru and S (from the photoactive SO_2_ ligand) in [Ru­(SO_2_)­(NH_3_)_4_ (pyridine)]­[tosylate]_2_; PDOS for (a) Ru and N (from *trans*-ligand 4-Cl-pyridine), as well as (b) Ru and S (from the photoactive SO_2_ ligand) in [Ru­(SO_2_)­(NH_3_)_4_(4-Cl-pyridine)]­[tosylate]_2_; PDOS for (a) Ru and N (from *trans*-ligand 4-Br-pyridine), as well as (b) Ru and S (from the photoactive SO_2_ ligand) in [Ru­(SO_2_)­(NH_3_)_4_(4-Br-pyridine)]­[tosylate]_2_; PDOS for (a) Ru and N (from *trans*-ligand 3-I-pyridine), as well as (b) Ru and S (from the photoactive SO_2_ ligand) in [Ru­(SO_2_)­(NH_3_)_4_(3-I-pyridine)]­[tosylate]_2_; PDOS for (a) Ru and N (from *trans*-ligand pyridine), as well as (b) Ru and S (from the photoactive SO_2_ ligand) in [Ru­(SO_2_)­(NH_3_)_4_(pyridine)]­Cl_2_·H_2_O; PDOS for (a) Ru and N (from *trans*-ligand 3-Cl-pyridine), as well as (b) Ru and S (from the photoactive SO_2_ ligand) in [Ru­(SO_2_)­(NH_3_)_4_(3-Cl-pyridine)]­Cl_2_·H_2_O; PDOS for (a) Ru and N (from *trans*-ligand 4-Cl-pyridine), as well as (b) Ru and S (from the photoactive SO_2_ ligand) in [Ru­(SO_2_)­(NH_3_)_4_(4-Cl-pyridine)]­Cl_2_·H_2_O; absorption spectra of [Ru­(SO_2_)­(NH_3_)_4_(H_2_O)]­[tosylate]_2_ generated by *turbo_lanczos* and *turbo_davidson* modules within QE; frontier orbitals that exist in [Ru­(SO_2_)­(NH_3_)_4_(3-F-pyridine)]­[tosylate]_2_; frontier orbitals that exist in [Ru­(SO_2_)­(NH_3_)_4_(3-Cl-pyridine)]­[tosylate]_2_; frontier orbitals that exist in [Ru­(SO_2_)­(NH_3_)_4_(3-Br-pyridine)]­[tosylate]_2_; transition energies, wavelengths, oscillator strengths, and electron–hole components for [Ru­(NH_3_)_4_(SO_2_)­(3-F-pyridine)]­[tosylate]_2_; transition energies, wavelengths, oscillator strengths, and electron–hole components for [Ru­(NH_3_)_4_(SO_2_)­(3-Cl-pyridine)]­[tosylate]_2_; transition energies, wavelengths, oscillator strengths, and electron–hole components for [Ru­(NH_3_)_4_(SO_2_)­(3-Br-pyridine)]­[tosylate]_2_ (PDF)
