# Supplementary material for: Dark- and Photoexcited-State Electronic Structures of [Ru(SO2)(NH3)4X]Y Complexes and Their Relation to SO2 Photoconversion Efficiency
Source: J Phys Chem Lett. 2026 Sep 24;17(40):11494–503. doi: 10.1021/acs.jpclett.6c02623 (PMC13656775; doi:10.1021/acs.jpclett.6c02623)
Supplement: Supplementary file 1 [file jz6c02623_si_002.pdf]

Supporting Information:

Dark- and Photoexcited-State Electronic Structures of $[Ru(SO_2)(NH_3)_4X]Y$ Complexes and Their Relation to SO_2 Photoconversion Efficiency

*Haoqian Zhou[1], and Jacqueline M. Cole[1, *]*

[1] Ray Dolby Centre, Cavendish Laboratory, Department of Physics, University of Cambridge, J. J. Thomson Avenue, Cambridge CB3 0US, U.K.

* Author for correspondence (J. M. Cole): jmc61@cam.ac.uk

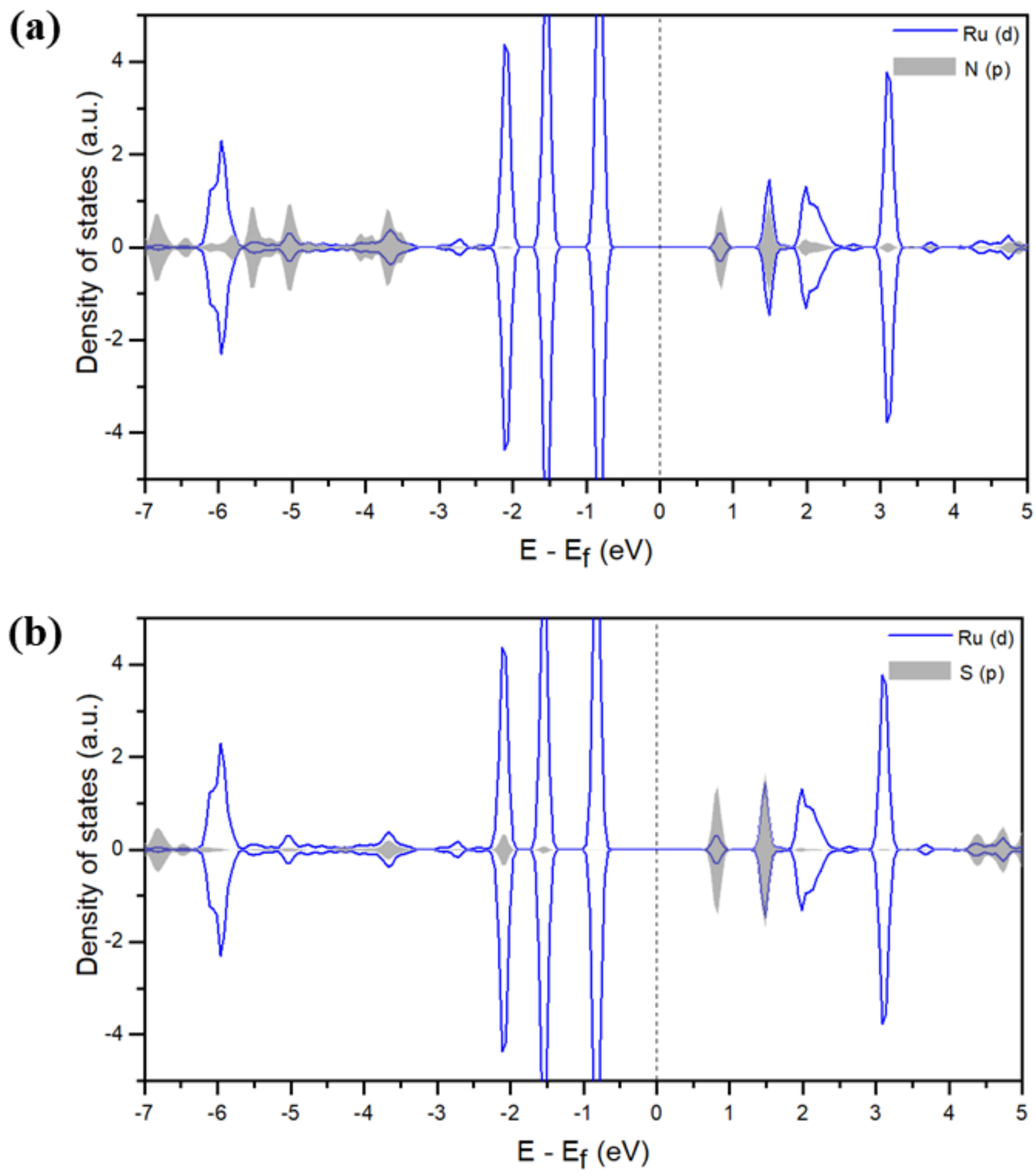

Figure S1. The projected density of states (PDOS) for (a) Ru and N (from *trans*-ligand 3-Cl-pyridine), as well as (b) Ru and S (from the photoactive SO_2 ligand) in $[Ru(SO_2)(NH_3)_4(3\text{-Cl-pyridine})][tosylate]_2$ (**3**). PDOS contributions from the ligand *p*-states are shown in grey; those of Ru *d*-states are given in blue. The dashed line indicates the Fermi level.

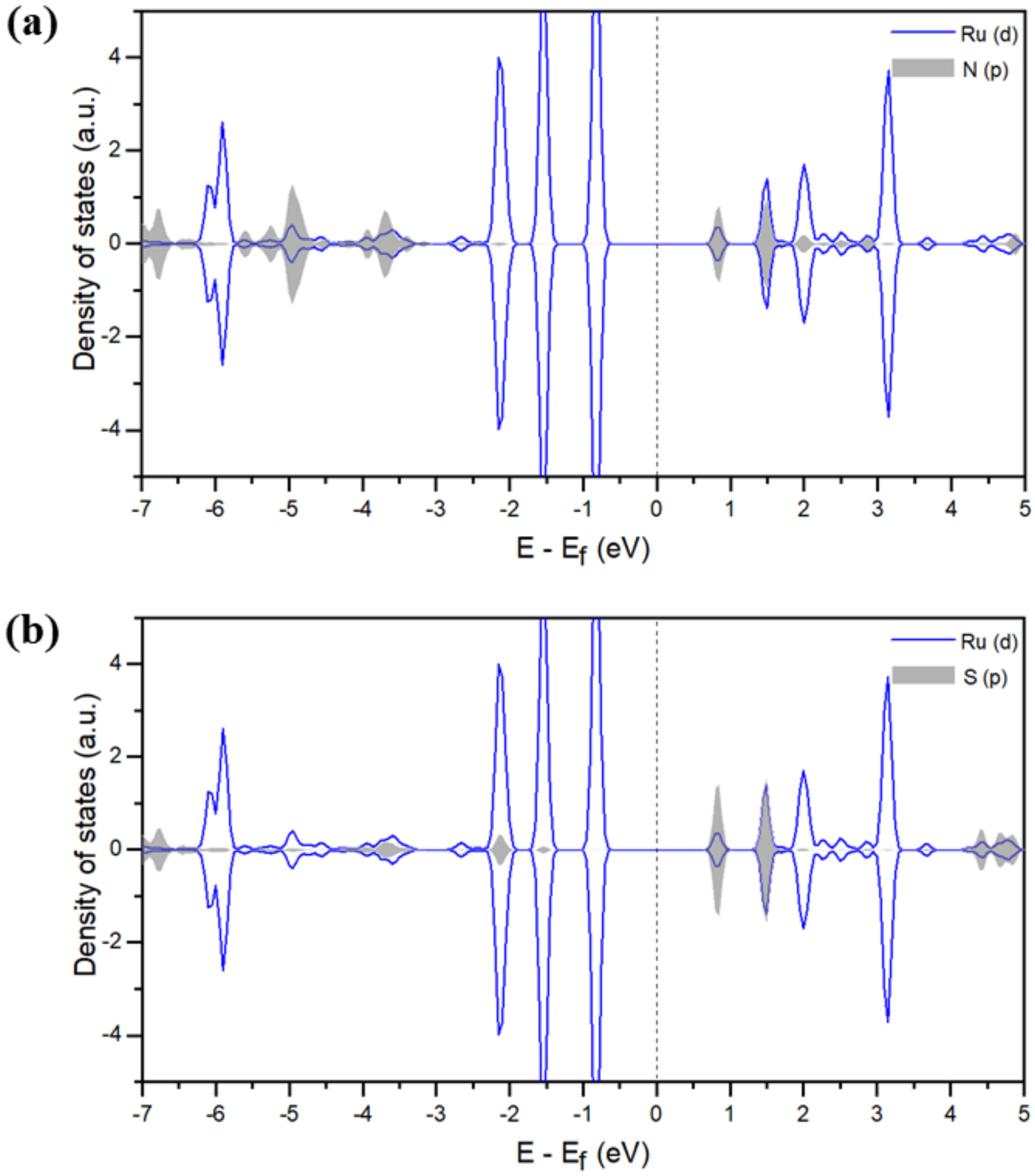

Figure S2. The projected density of states (PDOS) for (a) Ru and N (from *trans*-ligand 3-Br-pyridine), as well as (b) Ru and S (from the SO_2 ligand) in $[Ru(SO_2)(NH_3)_4$(3-Br-pyridine)][tosylate]$_2$ (**4**). PDOS contributions from the ligand *p*-states are shown in grey; those of Ru *d*-states are given in blue. The dashed line indicates the Fermi level.

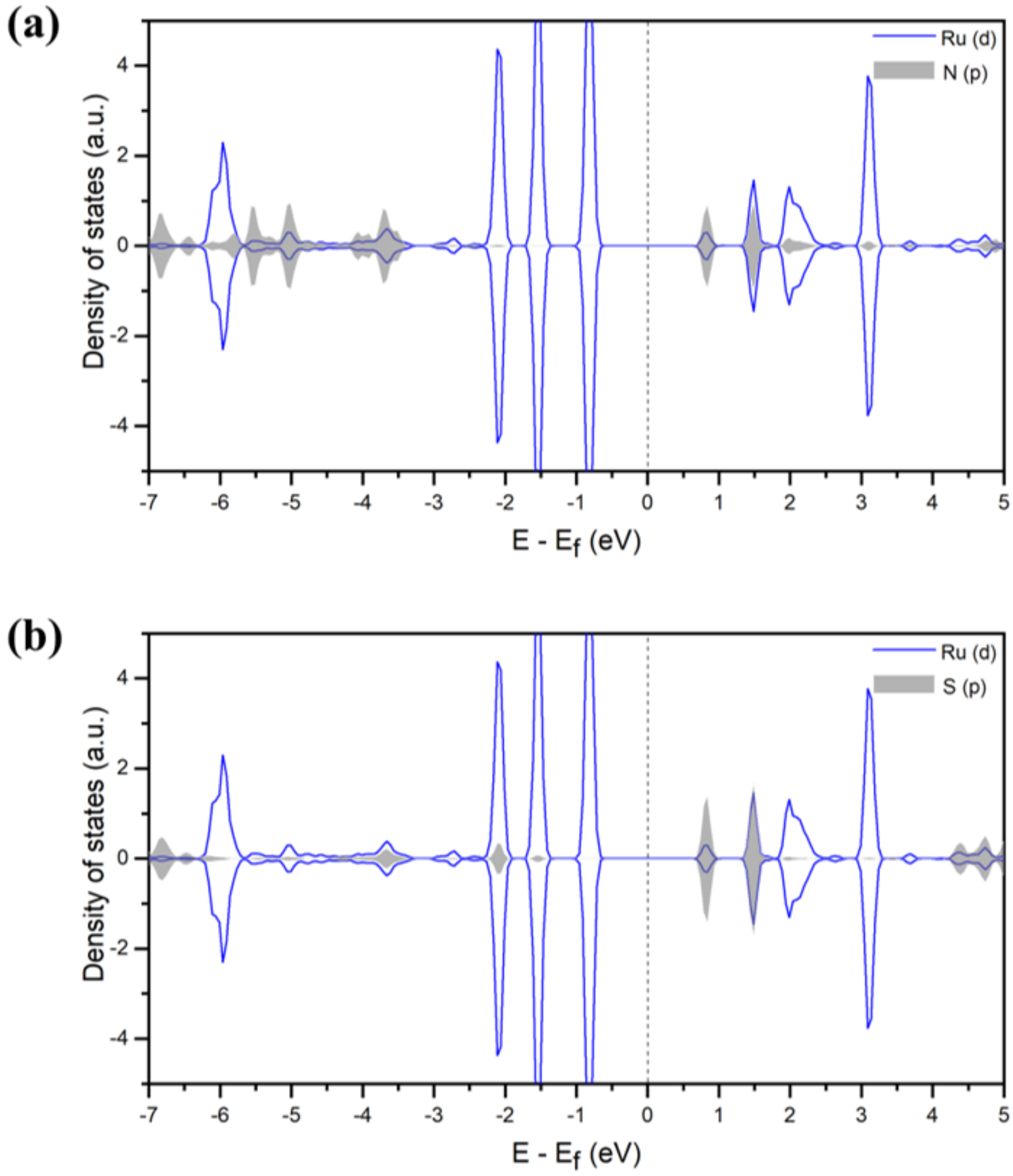

Figure S3. The projected density of states (PDOS) for (a) Ru and N (from *trans*-ligand pyridine), as well as (b) Ru and S (from the SO_2 ligand) in [Ru(SO_2)(NH_3)$_4$(pyridine)][tosylate]$_2$. PDOS contributions from the ligand *p*-states are shown in grey; those of Ru *d*-states are given in blue. The dashed line indicates the Fermi level.

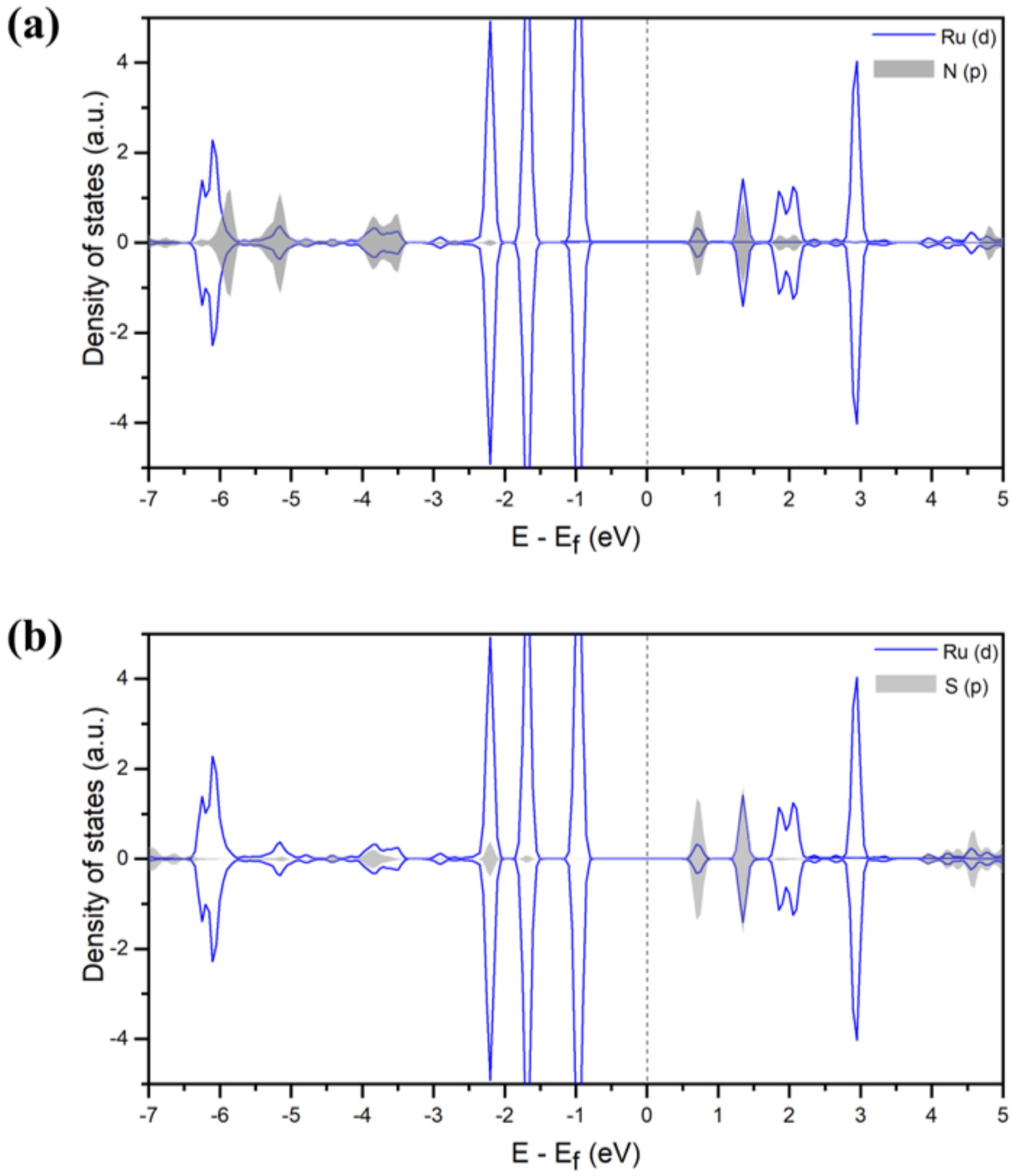

Figure S4. The projected density of states (PDOS) for (a) Ru and N (from *trans*-ligand 4-Cl-pyridine), as well as (b) Ru and S (from the SO_2 ligand) in $[Ru(SO_2)(NH_3)_4$(4-Cl-pyridine)][tosylate]$_2$. PDOS contributions from the ligand *p*-states are shown in grey; those of Ru *d*-states are given in blue. The dashed line indicates the Fermi level.

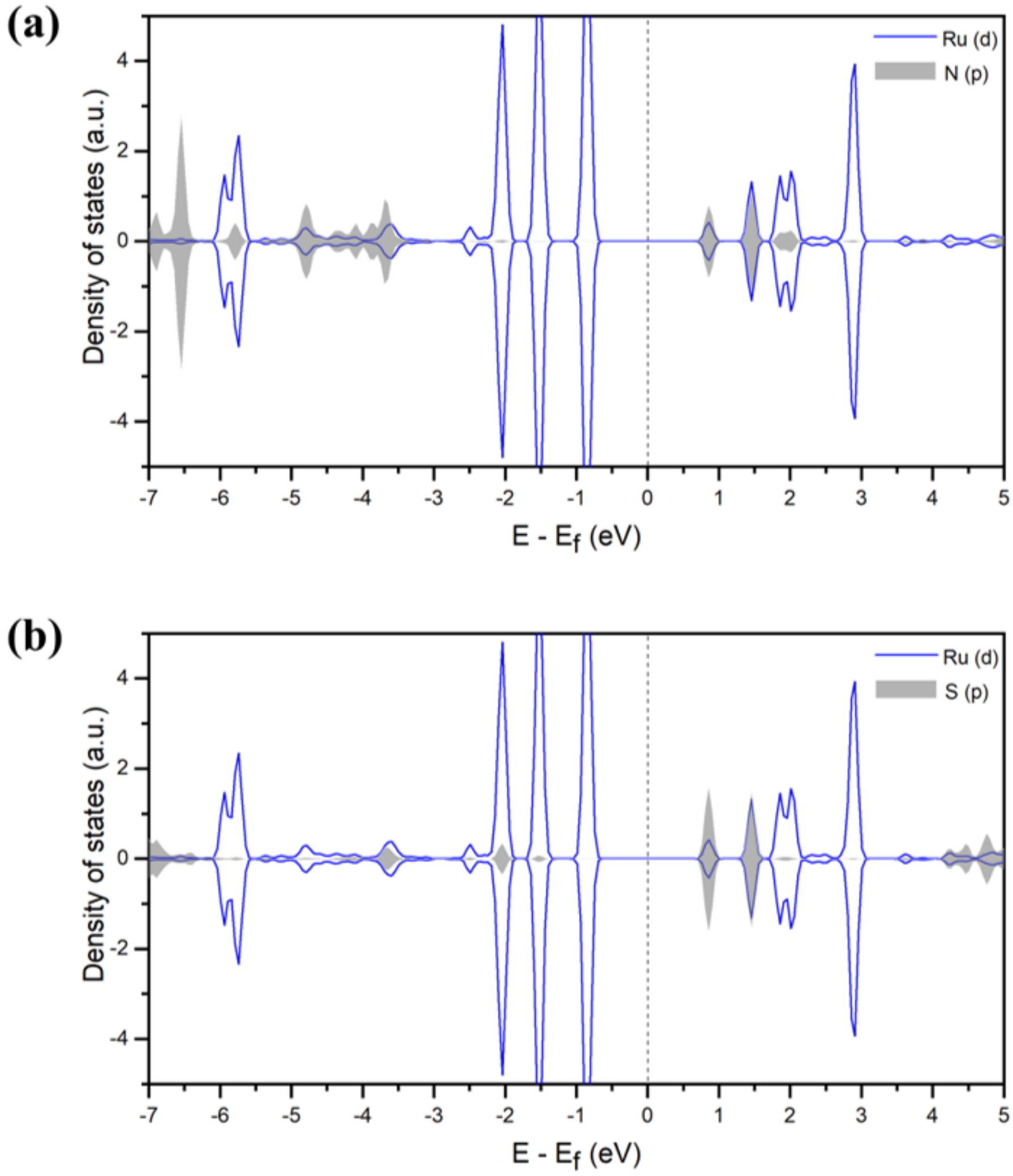

Figure S5. The projected density of states (PDOS) for (a) Ru and N (from *trans*-ligand 4-Br-pyridine), as well as (b) Ru and S (from the SO_2 ligand) in $[Ru(SO_2)(NH_3)_4(4\text{-Br-pyridine})][tosylate]_2$. PDOS contributions from the ligand *p*-states are shown in grey; those of Ru *d*-states are given in blue. The dashed line indicates the Fermi level.

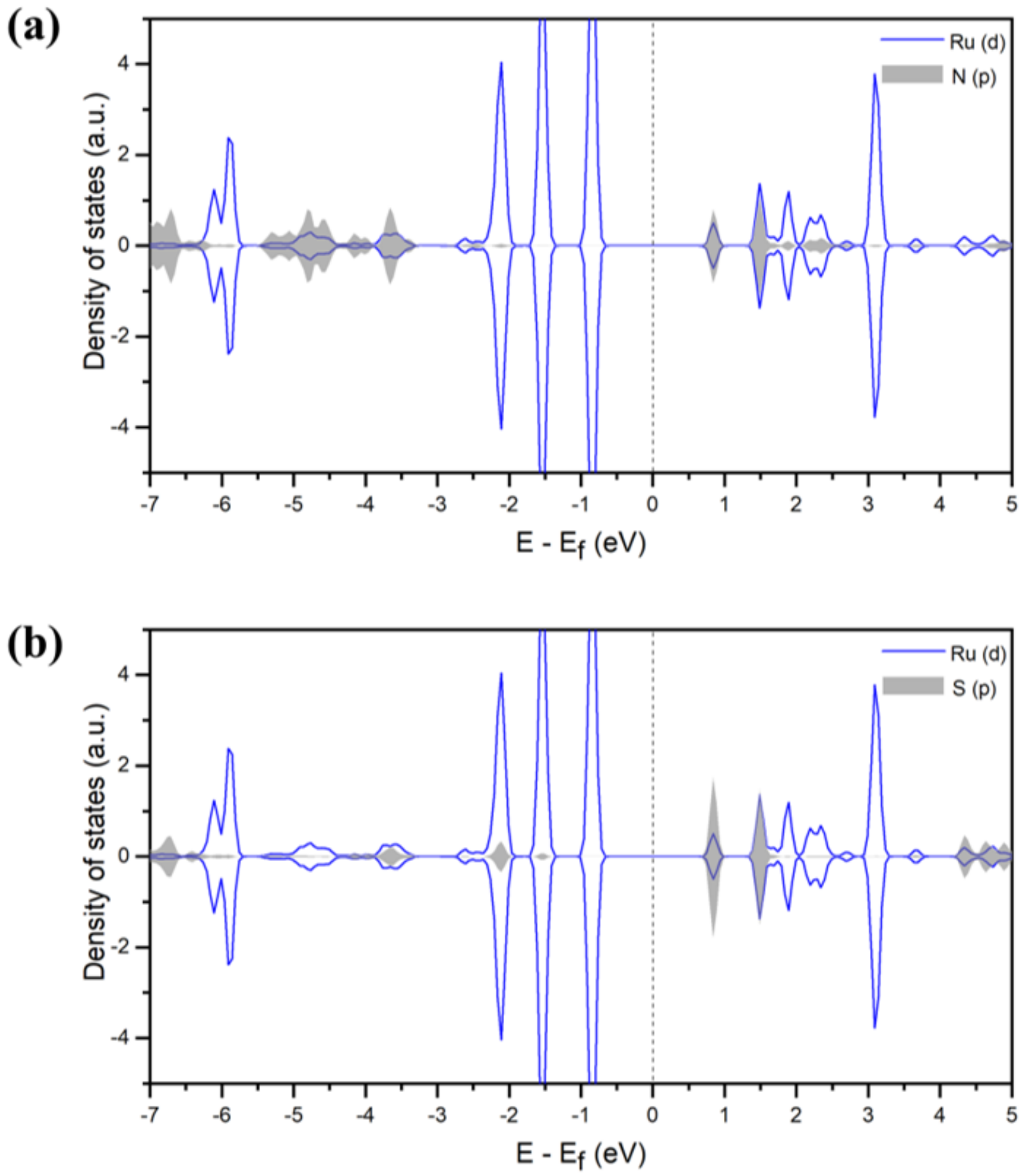

Figure S6. The projected density of states (PDOS) for (a) Ru and N (from *trans*-ligand 3-I-pyridine), as well as (b) Ru and S (from the SO_2 ligand) in [Ru(SO_2)(NH_3)$_4$(3-I-pyridine)][tosylate]$_2$. PDOS contributions from the ligand *p*-states are shown in grey; those of Ru *d*-states are given in blue. The dashed line indicates the Fermi level.

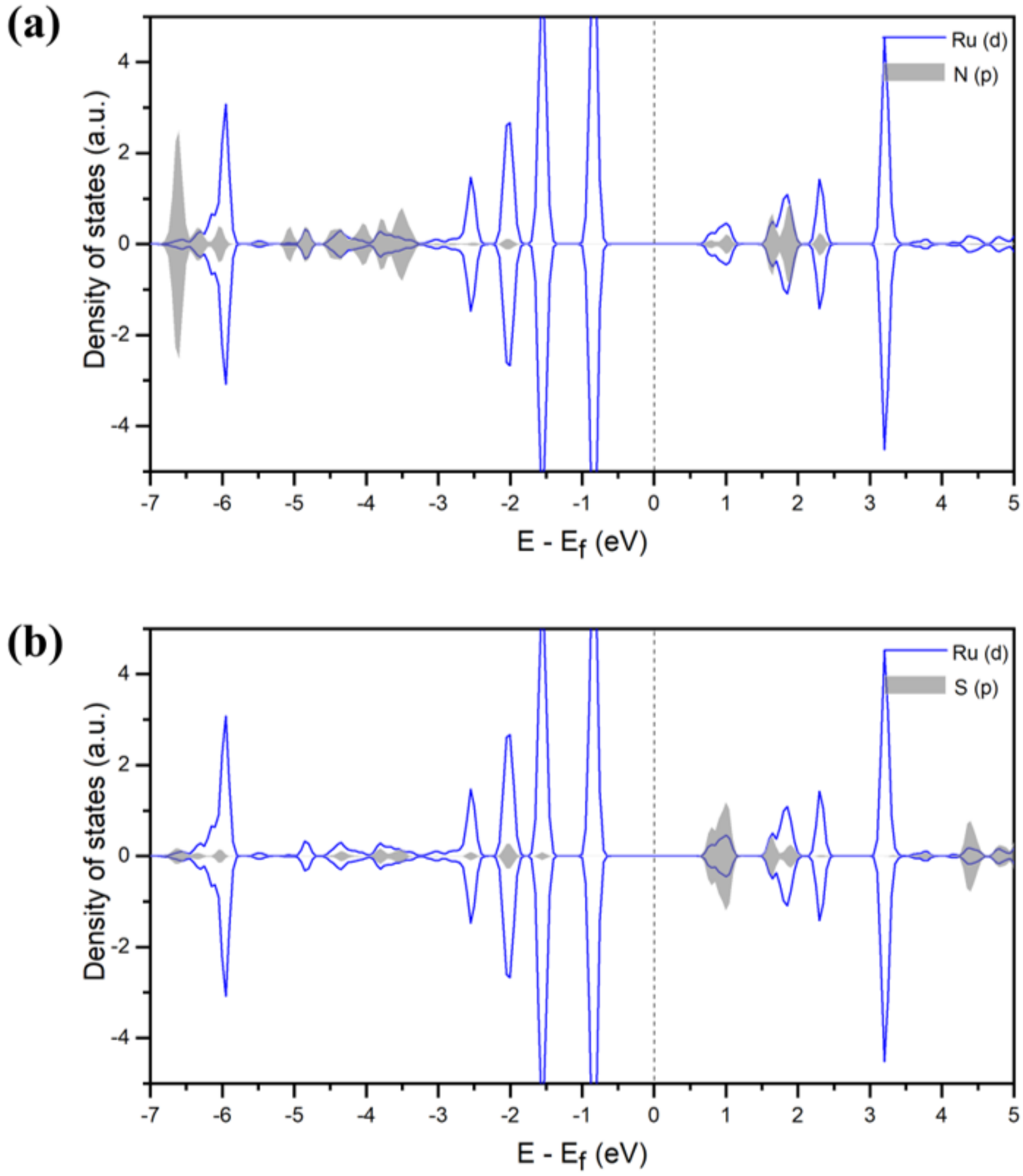

Figure S7. The projected density of states (PDOS) for (a) Ru and N (from *trans*-ligand pyridine), as well as (b) Ru and S (from the SO_2 ligand) in $[Ru(SO_2)(NH_3)_4(pyridine)]Cl_2\cdot H_2O$. PDOS contributions from the ligand *p*-states are shown in grey; those of Ru *d*-states are given in blue. The dashed line indicates the Fermi level.

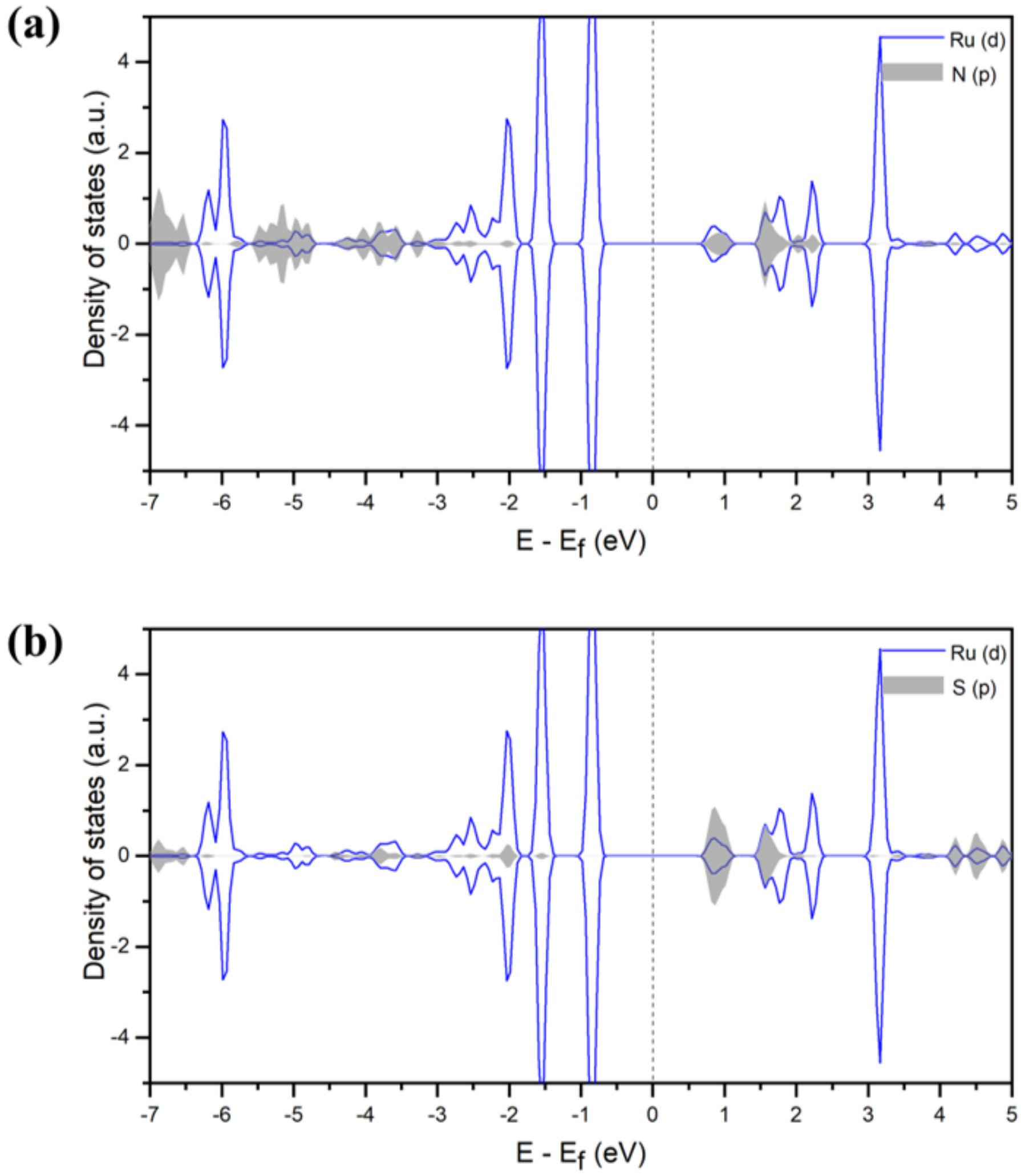

Figure S8. The projected density of states (PDOS) for (a) Ru and N (from *trans*-ligand 3-Cl-pyridine), as well as (b) Ru and S (from the SO_2 ligand) in $[Ru(SO_2)(NH_3)_4(3\text{-Cl-pyridine})]Cl_2\cdot H_2O$. PDOS contributions from the ligand *p*-states are shown in grey; those of Ru *d*-states are given in blue. The dashed line indicates the Fermi level.

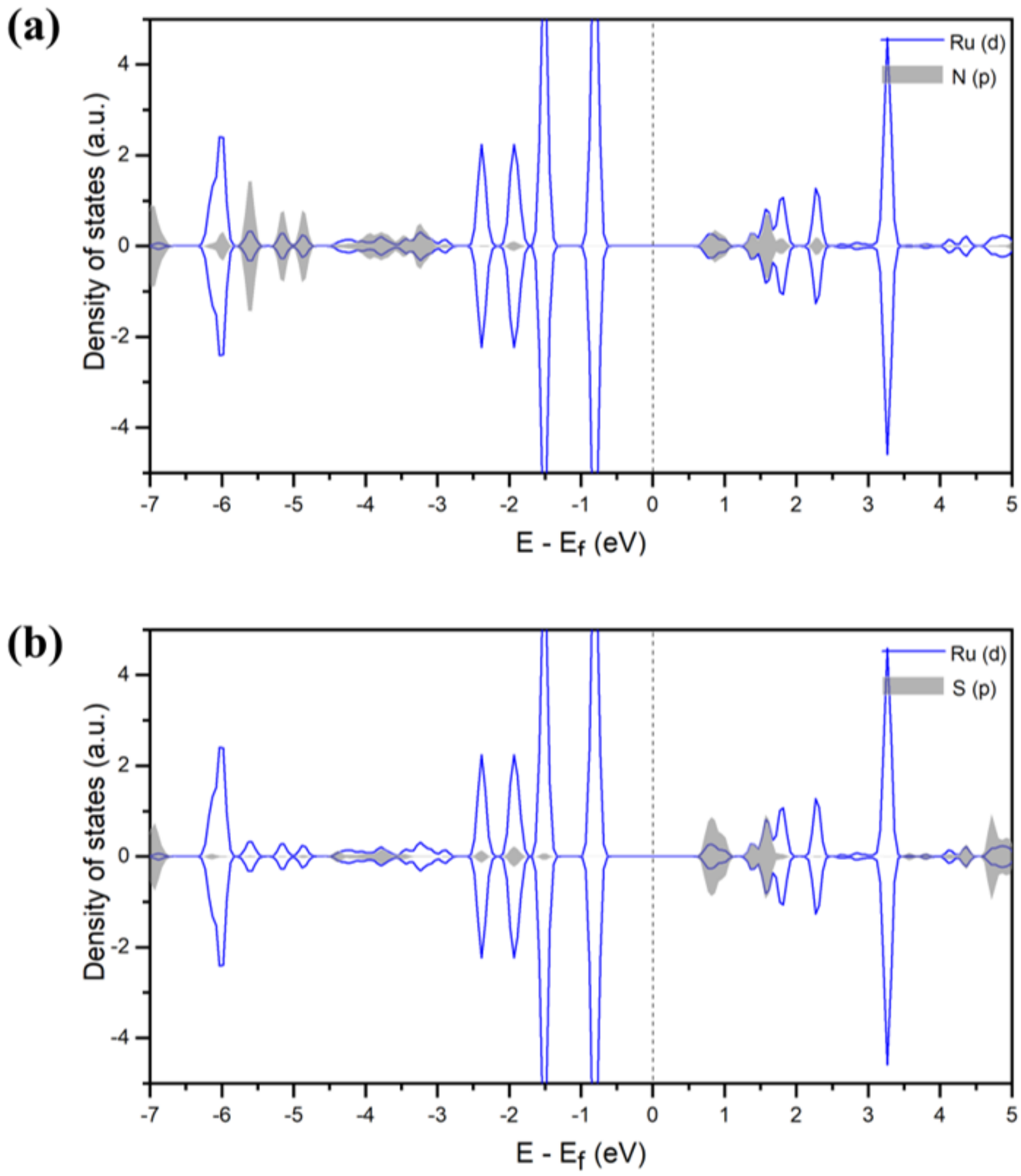

Figure S9. The projected density of states (PDOS) for (a) Ru and N (from *trans*-ligand 4-Cl-pyridine), as well as (b) Ru and S (from the SO_2 ligand) in $[Ru(SO_2)(NH_3)_4$(4-Cl-pyridine)$]Cl_2 \cdot H_2O$. PDOS contributions from the ligand *p*-states are shown in grey; those of Ru *d*-states are given in blue. The dashed line indicates the Fermi level.

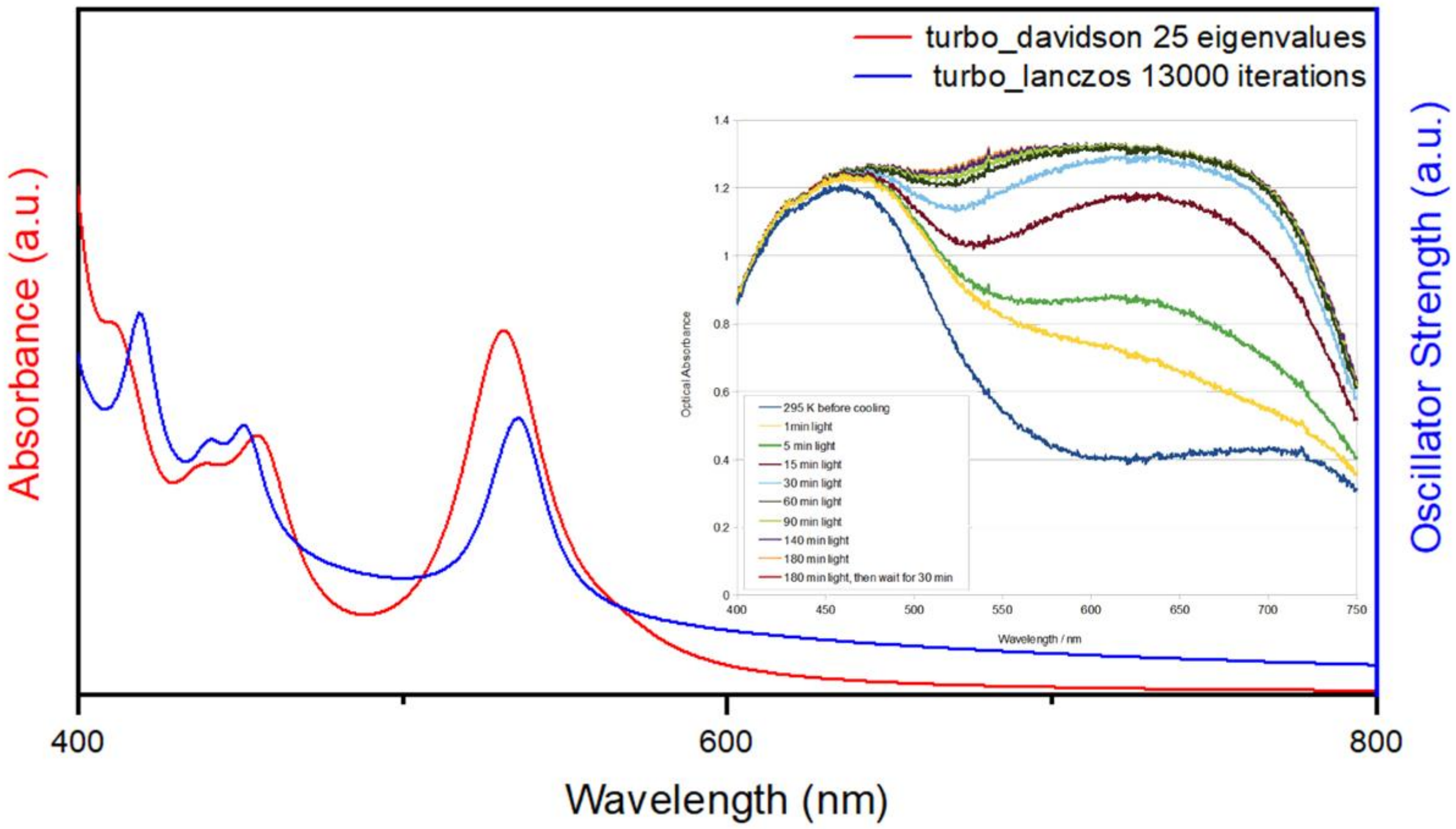

Figure S10. Absorption spectra of $[Ru(SO_2)(NH_3)_4(H_2O)][tosylate]_2$ (**1**) generated by *turbo_lanczos* and *turbo_davidson* modules within QE. Here, 13000 iterations and 25 eigenvalues were employed for the calculations using *turbo_lanczos* and *turbo_davidson* modules, respectively. The inset in the upper right panel displays the experimentally obtained absorption spectrum of **1** as presented in **Figure 2** of ref. 1 (reproduced with permission from the authors).

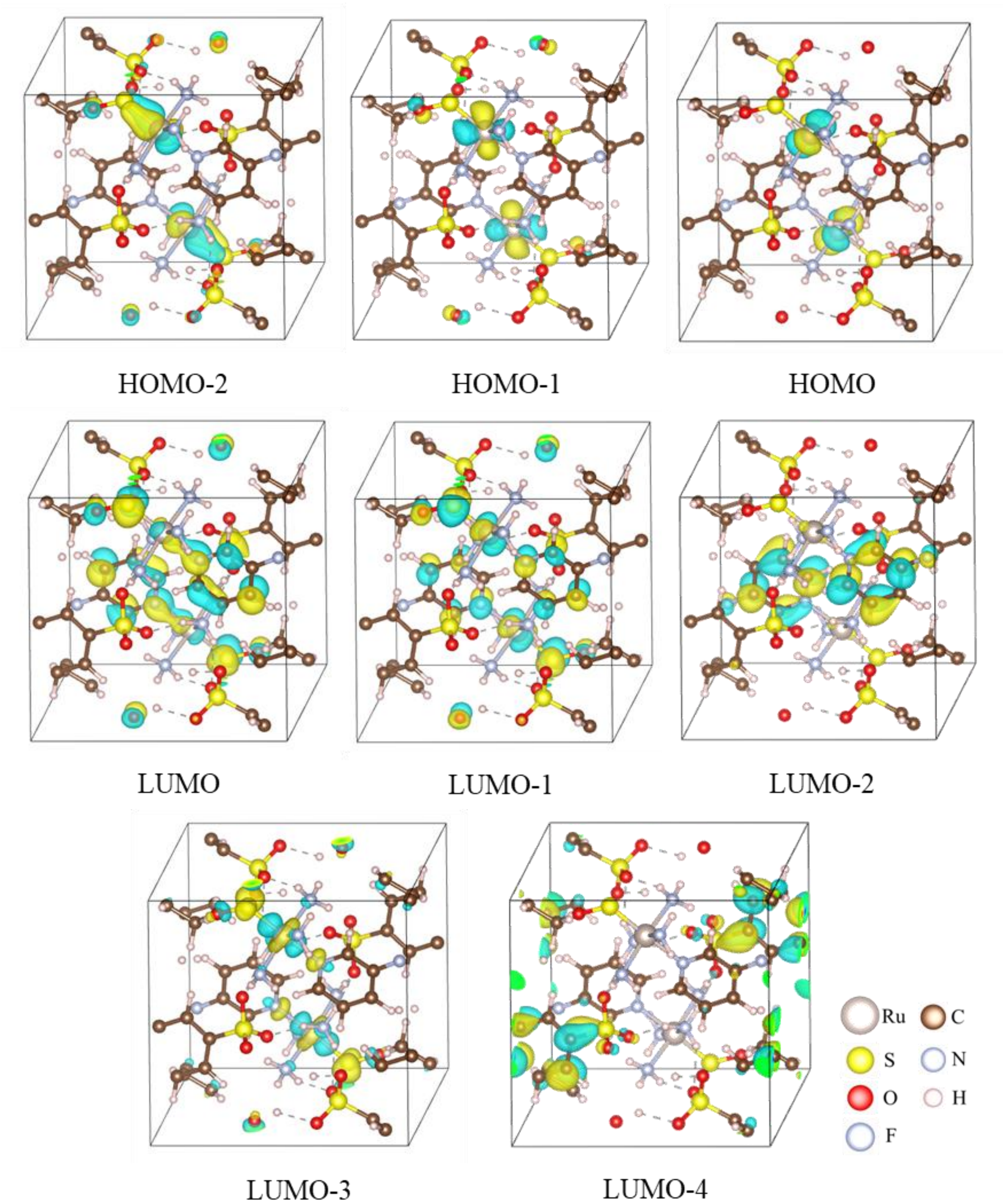

Figure S11. Isosurface (0.003 a.u.) of frontier orbitals that exist in [Ru(SO_2)(NH_3)$_4$(3-F-pyridine)][tosylate]$_2$ (**2**); The yellow and blue regions represent two different phases of the wavefunction.

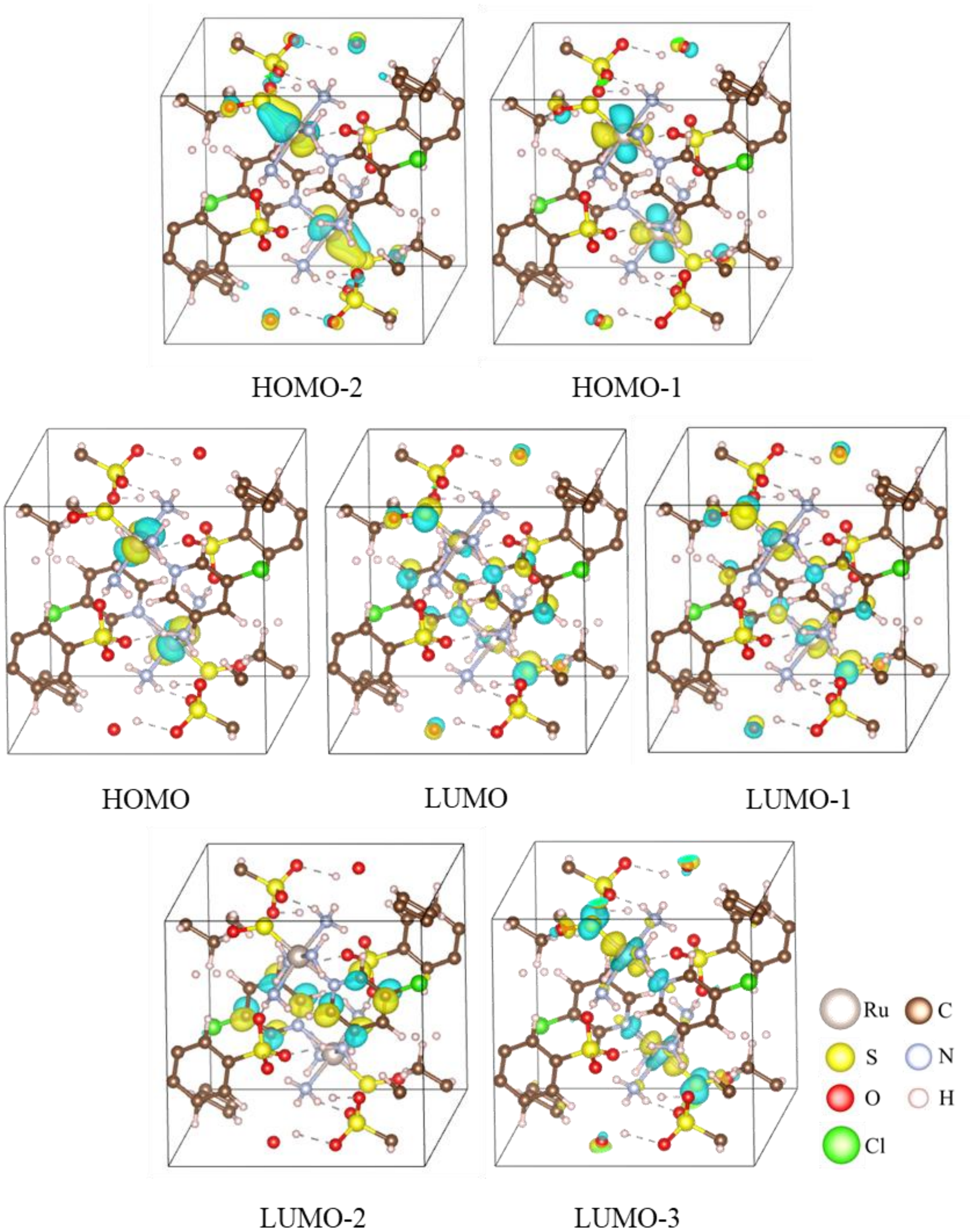

Figure S12. Isosurface (0.003 a.u.) of frontier orbitals that exist in [Ru(SO_2)(NH_3)$_4$(3-Cl-pyridine)][tosylate]$_2$ (**3**). The yellow and blue regions represent two different phases of the wavefunction.

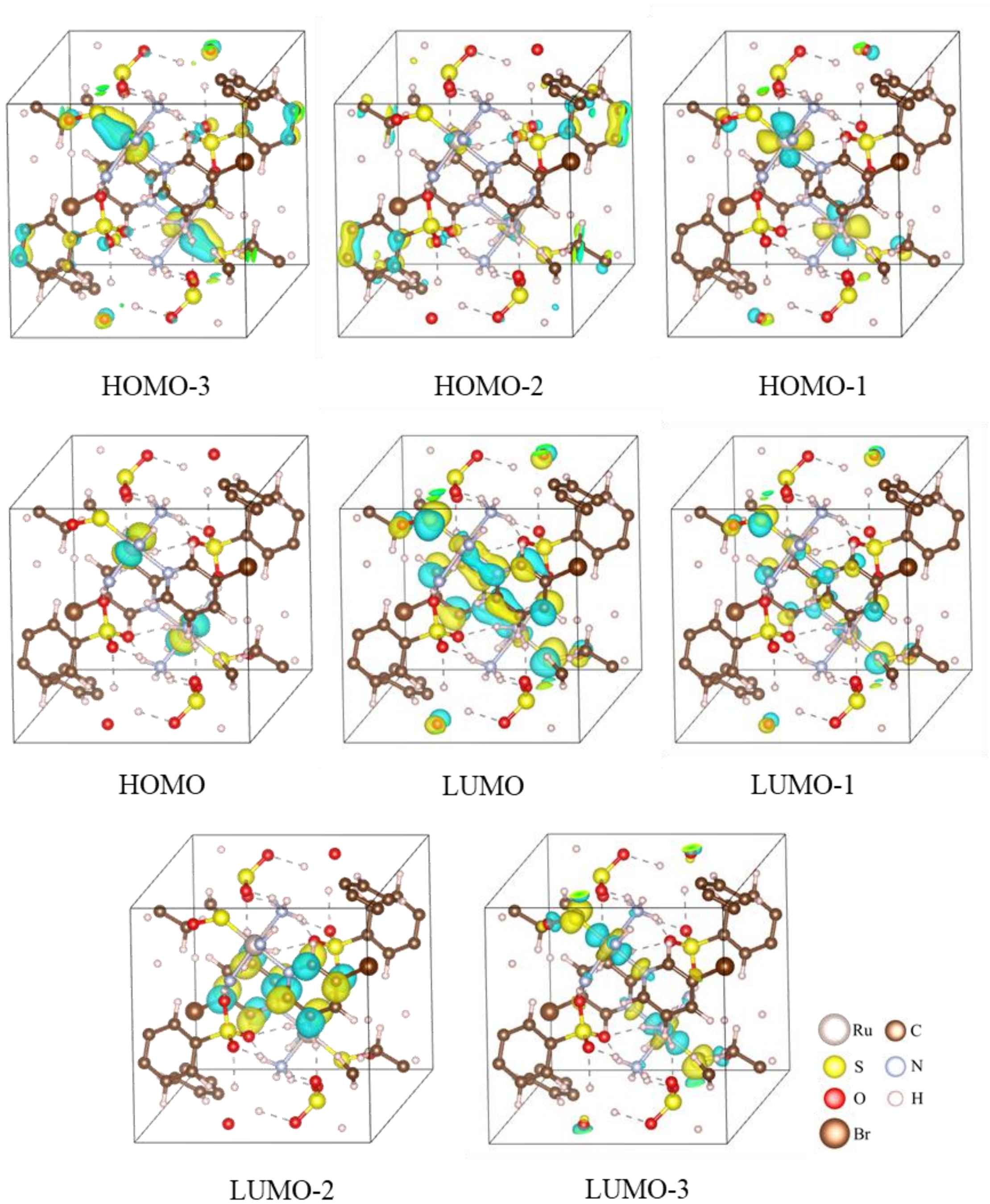

Figure S13. Isosurface (0.003 a.u.) of frontier orbitals that exist in $[Ru(SO_2)(NH_3)_4(3\text{-}Br\text{-}pyridine)][tosylate]_2$ (**4**). The yellow and blue regions represent two different phases of the wavefunction.

Table S1. Transition energies, wavelengths, oscillator strengths, and electron-hole components for $[Ru(NH_3)_4(SO_2)(3\text{-F-pyridine})][tosylate]_2$ (**2**) in the visible range (400-800 nm) of absorption. Only contributions ≥ 5% are reported.

Energy (eV)	Wavelength (nm)	Oscillator strength	Major contributions	
1.60	775	0.08	HOMO → LUMO	99.79%
2.27	546	0.20	HOMO → LUMO+1	99.95%
2.34	530	0.40	HOMO–1 → LUMO	99.76%
2.39	519	0.57	HOMO → LUMO+1	96.68%
2.40	517	0.57	HOMO–1 → LUMO	97.66%
2.64	470	0.18	HOMO → LUMO+2	98.45%
2.84	436	0.25	HOMO → LUMO+3<br>HOMO → LUMO+4	90.38%<br>8.86%
2.88	431	0.46	HOMO → LUMO+3	95.08%
2.91	426	0.55	HOMO–2 → LUMO	99.81%

Table S2. Transition energies, wavelengths, oscillator strengths, and electron-hole components for $[Ru(NH_3)_4(SO_2)(3\text{-Cl-pyridine})][tosylate]_2$ (**3**) in the visible range (400-800 nm) of absorption. Only contributions ≥ 5% are reported.

Energy (eV)	Wavelength (nm)	Oscillator strength	Major contributions	
1.63	761	0.05	HOMO → LUMO	99.78%
2.30	539	0.19	HOMO → LUMO+1	99.95%
2.35	527	0.34	HOMO–1 → LUMO	99.78%
2.42	513	0.47	HOMO–1 → LUMO<br>HOMO → LUMO+1	73.94%<br>24.60%
2.49	498	0.29	HOMO → LUMO+2	98.35%
2.84	436	0.21	HOMO–2 → LUMO+3	94.33%
2.91	425	0.47	HOMO–2 → LUMO	98.27%

Table S3. Transition energies, wavelengths, oscillator strengths, and electron-hole components for $[Ru(NH_3)_4(SO_2)(3\text{-Br-pyridine})][tosylate]_2$ (**4**) in the visible range (400-800 nm) of absorption. Only contributions ≥ 5% are reported.

Energy (eV)	Wavelength (nm)	Oscillator strength	Major contributions	
1.64	756	0.22	HOMO → LUMO	99.79%
2.42	512	0.64	HOMO-1 → LUMO	26.63%
			HOMO → LUMO+1	70.27%
2.50	496	0.41	HOMO → LUMO+2	98.19%
2.83	439	0.67	HOMO → LUMO+3	99.70%
2.89	429	1.36	HOMO-3 → LUMO	82.42%
			HOMO-2 → LUMO	15.43%

Table S4. Calculated Ru *d*-band centers (relative to the Fermi level), literature-reported photoconversion efficiencies to η[1]-OSO and η[2]-(OS)O and in total, and irradiation wavelengths (λ_{irr}) for the eleven $[Ru(SO_2)(NH_3)_4X]Y$ crystal structures.

Complex	*d*-band center (eV)	Photo-conversion to η^1-OSO (%)	Photo-conversion to η^2-(OS)O (%)	Photo-conversion Efficiency (%)	λ_{irr} (nm)	Reference
$[Ru(SO_2)(NH_3)_4$ pyridine]tosylate$_2$	-3.25	16.5(6)	31.6(11)	48.1(13)	425–650	Ref.2
$[Ru(SO_2)(NH_3)_4$(3-Cl-pyridine)]tosylate$_2$	-3.35	36.2(4)	6.9(6)	43.1(7)	425–650	Ref.2
$[Ru(SO_2)(NH_3)_4$(4-Cl-pyridine)]tosylate$_2$	-3.22	/	41(2)	41(2)	425–650	Ref.2
$[Ru(SO_2)(NH_3)_4$(3-Br-pyridine)]tosylate$_2$	-3.26	100	/	100	505	Ref.3
$[Ru(SO_2)(NH_3)_4(H_2O)]$ tosylate$_2$	-3.39	/	11.1(1)	11.1(1)	505	Ref.1
$[Ru(SO_2)(NH_3)_4$(4-Br-pyridine)]tosylate$_2$	-3.38	/	100	100	505	Ref.4
$[Ru(SO_2)(NH_3)_4$pyridine]$Cl_2{\cdot}H_2O$	-3.14	44.5(7)	13.2(9)	57.7(11)	425–650	Ref.5
$[Ru(SO_2)(NH_3)_4$(3-Cl-pyridine)]$Cl_2{\cdot}H_2O$	-2.98	10.4(1)	/	10.4(1)	425–650	Ref.5
$[Ru(SO_2)(NH_3)_4$(4-Cl-pyridine)]$Cl_2{\cdot}H_2O$	-3.08	/	18.2(7)	18.2(7)	425–650	Ref.5
$[Ru(SO_2)(NH_3)_4$(3-I-pyridine)]tosylate$_2$	-3.27	100	/	100	505	Ref.6
$[Ru(SO_2)(NH_3)_4$(3-F-pyridine)]tosylate$_2$	-3.29	70.3(6)	/	70.3(6)	505	Ref.7

REFERENCES

(1) Cole, J. M.; Gosztola, D. J.; Sylvester, S. O. Low-energy optical switching of SO_2 linkage isomerisation in single crystals of a ruthenium-based coordination complex. *RSC Adv.* **2021**, *11* (22), 13183-13192.

(2) Sylvester, S. O.; Cole, J. M.; Waddell, P. G.; Nowell, H.; Wilson, C. SO_2 phototriggered crystalline nanomechanical transduction of aromatic rotors in tosylates: Rationalization via photocrystallography of [Ru $(NH_3)_4SO_2X$]tosylate$_2$ (X= pyridine, 3-Cl-pyridine, 4-Cl-pyridine). *J. Phys. Chem. C* **2014**, *118* (29), 16003-16010.

(3) Cole, J. M.; Gosztola, D. J.; Velazquez-Garcia, J. d. J. Nanooptomechanical transduction in a single crystal with 100% photoconversion. *J. Phys. Chem. C* **2021**, *125* (16), 8907-8915.

(4) Cole, J. M.; Gosztola, D. J.; Velazquez-Garcia, J. d. J.; Guest, J. R. Ternary molecular switching in a single-crystal optical actuator with correlated crystal strain. *Nat. Commun.* **2025**, *16* (1), 1546.

(5) Sylvester, S. O.; Cole, J. M.; Waddell, P. G. Photoconversion bonding mechanism in ruthenium sulfur dioxide linkage photoisomers revealed by in situ diffraction. *J. Am. Chem. Soc.* **2012**, *134* (29), 11860-11863.

(6) Cole, J. M.; Gosztola, D. J.; de J. Velazquez-Garcia, J.; Wang, S. G.; Chen, Y. S. Single-crystal optical actuation generated by 100% SO_2 linkage photoisomerization in a ruthenium-based coordination complex. *J. Phys. Chem. C* **2021**, *125* (36), 20059-20066.

(7) Cole, J. M.; Gosztola, D. J.; Velazquez-Garcia, J. d. J. Structural capture of η^1-OSO to η^2-(OS) O coordination isomerism in a new ruthenium-based SO_2-linkage photoisomer that exhibits single-crystal optical actuation. *J. Phys. Chem. C* **2022**, *126* (13), 6047-6059.
